# The Aloe Family recipe for open and specialized healthcare LLMs

**DOI:** 10.1038/s41746-026-02637-y

**Published:** 2026-05-11

**Authors:** Dario Garcia-Gasulla, Jordi Bayarri-Planas, Ashwin Kumar Gururajan, Enrique Lopez-Cuena, Adrian Tormos, Daniel Hinjos, Pablo Bernabeu-Perez, Anna Arias-Duart, Pablo Agustin Martin-Torres, Marta Gonzalez-Mallo, Sergio Alvarez-Napagao, Eduard Ayguadé-Parra, Ulises Cortés

**Affiliations:** 1 Barcelona Supercomputing Center (BSC-CNS), Barcelona, Spain; 2 Universitat Politècnica de Catalunya - Barcelona Tech (UPC), Barcelona, Spain

**Keywords:** Computational models, Machine learning

## Abstract

The growing interest in the application of Large Language Models (LLMs) for healthcare comes with a demand for better open-source LLMs, and stronger reassurances regarding their performance. To advance in this direction, this work conducts a thorough and transparent study of LLM model training and benchmarking in healthcare, releasing as open assets all resources needed for reproducing the Aloe models and its results (weights, data and code). This includes details on optimized data preprocessing and training, combining curated public data with synthetic samples for a total of 1.8B training tokens; enhanced safety, induced through Direct Preference Optimization (DPO), aligning Aloe models for ethical robustness and against jailbreaking attacks; and finally model performance, evaluated thoroughly through close-ended, open-ended, safety, and human assessments. To boost inference efficacy and test the upper bounds of open LLM performance, Aloe models are integrated with a Retrieval-Augmented Generation (RAG) system. The resultant models deliver competitive performance across healthcare benchmarks and medical fields while significantly improving safety and bias resilience. Model weights are released for research-only purposes, together with training and evaluation datasets, and RAG inference code. To enable the responsible release of such technology, this work is supported by a detailed healthcare-specific risk assessment. Building on top of base models like Llama 3.1 and Qwen 2.5, the Aloe models and their development recipe set a high standard for open-source medical LLMs, balancing top-tier performance with high ethical requirements.

## Introduction

A race has been going on for the last few years within the field of large language models (LLMs). One between open and closed models, between models that are examinable, tunable and free to use (Llama, Mistral, Qwen, DeepSeek), and models that are closed, private and inaccessible (GPT, Gemini, Claude, Grok). In such a race, particularly for domains like human healthcare where universal access is a fundamental right, it is essential that open models match the pace of closed alternatives. Good, competitive open models increase the standards for reliability, accessibility, and oversight in a field where safety requirements must be maximal. Competitive open models ensure that desirable applications of LLMs in healthcare (i.e., automating redundant tasks, reducing training costs, facilitating access to medical information) benefit everyone while upholding transparency and accountability.

In this paper we introduce the Aloe Family of open healthcare LLMs and a reproducible recipe for building, aligning, and deploying them. The recipe consists of five stages:

1. A large-scale domain corpus curation and synthetic chain-of-thought (CoT) data generation.
2. A supervised fine-tuning (SFT) to induce instruction-following capabilities and domain-specific knowledge.
3. A model merging step used to combine the strengths from diverse model versions.
4. A preference alignment using Direct Preference Optimization (DPO) to increase safety and adherence to human standards.
5. A retrieval-augmented inference (RAG) for boosting performance in deployment.

This work presents quantitative and qualitative evaluations (MCQA, open-ended generation, clinician preference trials, and adversarial safety tests) that map directly to these stages. Four healthcare-oriented LLMs (7B, 8B, 70B, 72B) are trained following this recipe, and their release is complemented by a focused healthcare-specific risk assessment, as well as practical recommendations for their mitigation.

Today’s most effective mechanism to build powerful healthcare LLMs is to fine-tune highly competitive generalist *pre-trained* models. These models already possess a strong capabilities in general language processing and generation, making them ideal targets for domain-specific fine-tuning. Through this process, fine-tuned models boost their performance at medical tasks, tailoring its outputs to healthcare applications. The alternative, pre-training a healthcare *base model from scratch*, would require the introduction of massive amounts of data outside of the healthcare field, increasing all expenses (human and compute hours) with very limited returns.

**Fig. 1:**
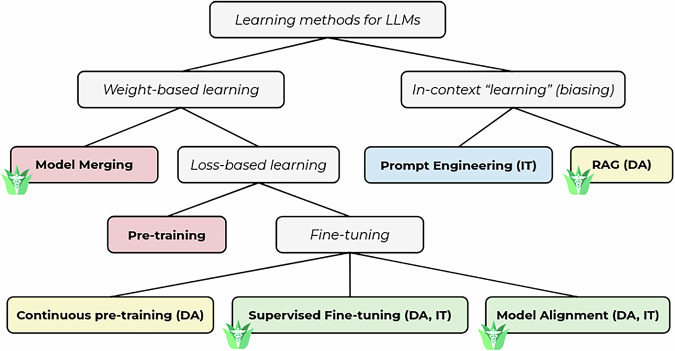
Summary of LLM training stages and their relations. In gray, general categories. In blue, methods for instruct tuning (IT). In yellow, methods for domain adaptation (DA). In green, methods for both IT and DA. In pink, other methods. The Aloe logo marks those techniques used on the Aloe Family.

When building a healthcare specific LLM on top of a pre-trained model, one may apply *continuous pre-training*, performing autoregressive learning on large amounts of arbitrarily structured, domain-specific data. This adjusts the content of the responses but not their style. To achieve a conversational model, *instruct tuning* or assistant adaptation is needed, which trains the model to answer user questions and requests. This can be done in domain-specific or general-purpose data. Notice instruct tuning is often considered a *supervised fine-tuning* (SFT) method since data used for training are curated QA pairs, and learning is only performed in the answer tokens. Beyond SFT, a third type of LLM tuning mechanism is *model alignment* (MA), which drives the model towards producing a preferable type of outputs with higher probability (e.g., RLHF, DPO). This is typically performed as a post-training stage to polish answer styles. Beyond loss-based learning entirely, there are other methods that can improve model performance. This includes *model merging*, an ensemble technique that changes the internal weights of the model by combining those of different variants following a set of heuristics. There’s also prompting strategies, sometimes called *in-context learning*, which has evolved into retrieval-augmented generation (RAG) for boosting the performance of models during inference through contextualization and input *bias*. All these options are available in LLM training and deployment, as are illustrated in Fig. 1.

The goal of this work is to contribute and advance the domain of open healthcare LLMs, providing a coherent, efficient and reproducible training strategy, while conducting an exhaustive and complementary evaluation. To that end, this work optimizes and combines four different types of learning (SFT, MA, RAG and merging), discarding others because of their prohibitive cost (pre-training), their lack of effectiveness (continuous pre-training) or their inconsistency (prompt engineering). Upon reviewing previous attempts within the healthcare domain in this section, and their struggles to outperform generalist models, this work proposes a reproducible strategy that can be competitive with private alternatives when boosted with RAG components, and safer than most alternatives (see Section Results). The result of this exercise is the Aloe Family of models, a set of specialized and open LLMs for the healthcare domain. This includes four models (7B, 8B, 70B and 72B) based on two different pre-trained sources (Llama 3.1 and Qwen 2.5). These are domain-specific (healthcare specialists), instruct-tuned (useful assistants) and aligned with human preferences (safer for users). All datasets used for training, including those curated and expanded, are shared for the community to use. All evaluation methods conducted (close-ended, open-ended, by medical speciality and model safety) are public and reusable, with the only exception of human experts preferences. Aloe Beta models are released with a healthcare-specific risk assessment for safe deployment. The relevance of all these efforts are discussed in Section “Discussion”.

**Fig. 2:**
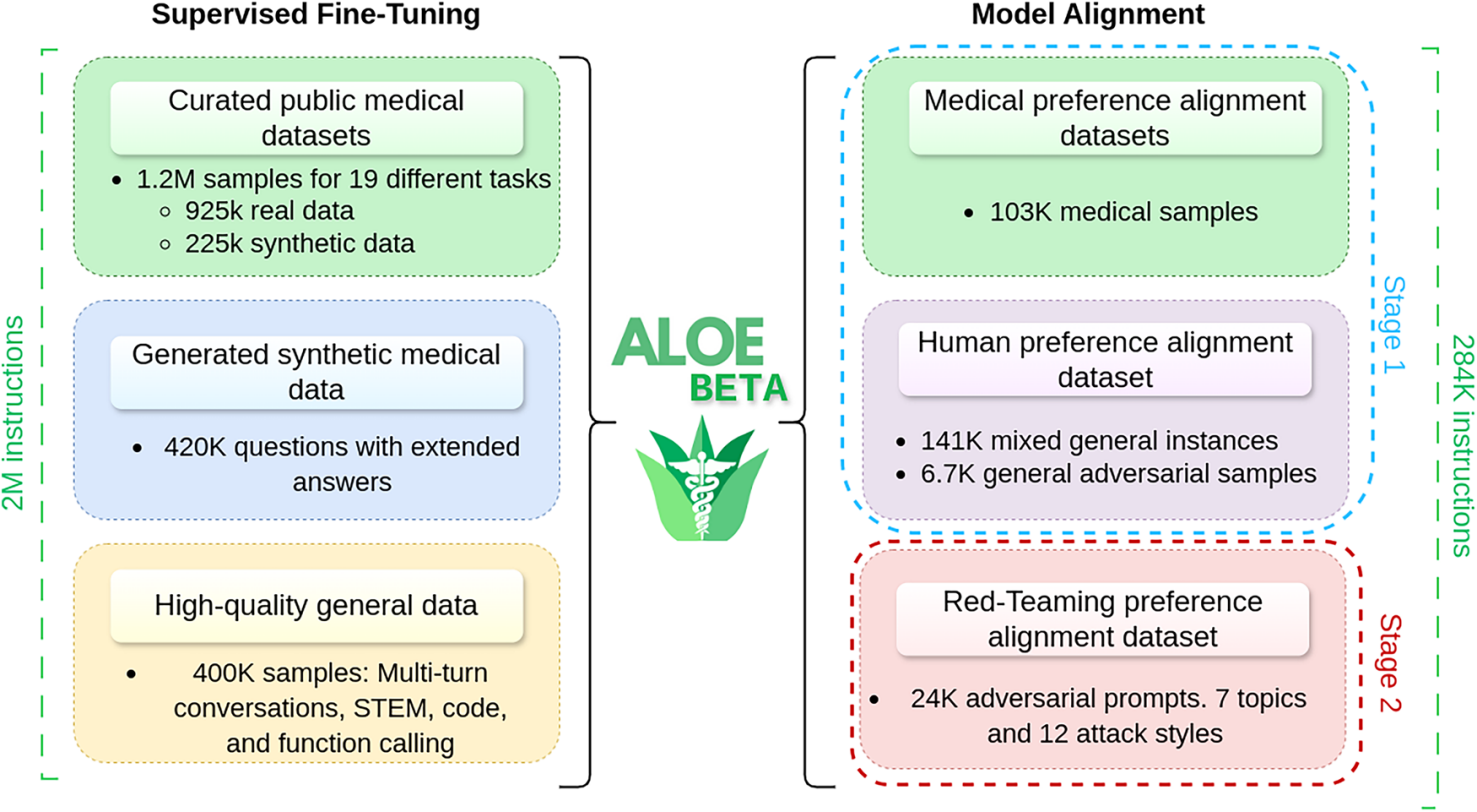
Summary of the data and training steps proposed in the Aloe recipe. On the left, data for the first, supervised-fine-tuning training stage. On the right, data for the second, model alignment training stage.

Training an LLM (even post-training one) is a huge undertaking that involves a lot of decisions related with the many different aspects of model development. In Section “Methods” this work reports extensive details on the training data (Section Training Data) and on the learning stages (Section Learning Stages) used to produce Aloe. By publishing all the assets and thoroughly reporting on those, the Aloe Family becomes a building block for the open healthcare LLM community, allowing for easily building on top of its models, improving or adapting them further for specific applications. An overview of the proposed recipe is shown in Fig. 2. A summary of the contributions is as follows:

- Release of four open healthcare specific, safety aligned LLMs (7B, 8B, 70B, 72B).
- Release of a 1.8B token training corpus, together with 400k general SFT samples.
- A complete preference alignment pipeline, including 276k samples.
- Unified recipe for model merging, DPO safety alignment and RAG model deployment.
- Human expert evaluation on open models for real world healthcare questions
- A healthcare-specific model risk assessment

Healthcare LLMs have seen significant advancements in the last few years, with private models claiming top performance in benchmarks using advanced prompt strategies (e.g., GPT-4 with Medprompt^1^ and MedPalm-2^2^). These models were recently joined by Med-Gemini^3^, built on top of Gemini 1.0 and 1.5 models, which introduces multimodal and web search functionalities. Unfortunately, all these private options remain inaccessible to the broader research community, limiting the capacity for developing and evaluating open healthcare LLMs.

In parallel, open models for healthcare have made substantial progress, using a wide range of architectures and strategies for improving performance in medical domains. MedAlpaca^4^, released in April 2023, is based on LLaMA-2 7B and 13B and instruct-tuned on a dataset containing 150,000 question-answer (QA) pairs. PMC-LLaMA^5^, introduced in May 2023, fine-tunes LLaMA-2 through continuous pre-training on a mix of books and papers, followed by instruct tuning on QA pairs. Similarly, Meditron, launched in November 2023, leverages continuous pre-training and fine-tuning on LLaMA-2 using a substantial dataset of medical papers, abstracts, and guidelines (48 billion tokens). Meditron^6^ includes a 7B and a 70B version and is tailored to specific benchmarks through targeted instruct tuning. The landscape expanded in 2024 with MMed-LLM-2^7^, a 7B model released in February 2024 trained on InternLM-2 using medical data from multilingual datasets and textbooks (25 billion tokens). MMed-LLM-2 excels in multilingual medical QA tasks, achieving state-of-the-art performance for languages such as Japanese and Chinese in its custom benchmark, MMedBench. BioMistral^8^, introduced the same month, focuses on continuous pre-training of medical papers on top of the instruct-tuned Mistral-7B. OpenBioLLM^9^ launched in April was designed specifically for the biomedical domain. Although its multiple-choice QA performance is reportedly strong, the training data and technical report remain undisclosed. In the same month, Aloe Alpha^10^ was introduced as the first iteration of the Aloe family, including techniques like merging and red teaming. Built on Mistral and LLaMA-3, Aloe Alpha leveraged public datasets enhanced with synthetic Chain of Thought (CoT) data and applied Direct Preference Optimization for alignment. This 7B model set a new standard for ethical performance among open healthcare LLMs, with evaluations covering bias, toxicity, and risk. Shortly after, Ultramedical^11^ was released, together with a suite of high-quality manual and synthetic biomedical datasets called UltraMedical Collections. These datasets, featuring preference annotations across various advanced LLMs, are used to fine-tune specialized medical models based on LLaMA-3, achieving remarkable results on diverse medical benchmarks. In August, Med42-v2^12^, built on LLaMA-3, employed a two-stage training process, instruction fine-tuning and preference alignment, to address clinical queries effectively. Finally, by December 2024, HuatuoGPT-o1^13^ introduced a novel reasoning-focused training recipe, using 40,000 verifiable medical problems to enhance LLM reasoning capabilities in under-explored domains like medicine.

Of all these recently released healthcare LLMs, only a few include full data and training details, which limits their reproducibility and accessibility. Even less include a safety alignment phase, limiting their reliability in deployment domains. See Table 1 for details. Aloe, by being more comprehensive and transparent than all of the alternatives, fills in this gap. Beyond healthcare LLMs, the other main baseline are general purpose LLMs, which are often highly reliable for healthcare tasks even without a domain adaptation phase. For this reason, this work includes the instruct versions released by the original authors as a priority baseline.

**Table 1:** Comparison of medical LLM features

	

Green: Yes, Red: No. Yellow Min: Minimal. Pre-training includes continuous pre-training. Jailbreak Prot: Protection against jailbreaking

Beyond the technical development of LLMs for healthcare, the field needs to advance as well on the identification of associated risks and ethical considerations. A few works have explicitly reviewed the potential harms and risks of this technology in such a sensitive domain^14–16^, including challenges of bias, toxicity, sycophancy, and hallucinations in medical applications^17^. Tackling these issues remains imperative as open healthcare LLMs continue to evolve and strive for parity with private models.

The summary of the main features characterizing these related models, together with an overview of the contributions of the Aloe Family models are shown in Table 1. Notice the most distinctive features of Aloe being model merging, jailbreaking protection, RAG integration, model risk assessment and human expert evaluation.

## Results

The relevance of the Aloe Family models is defined by their capacity to safely serve users in a variety of domains. To assess such models coherently, evaluation must be equally varied. However, even when using a large set of benchmarking tasks, drawing reliable conclusions remains challenging^18,19^. As a result, the question on how to assess the generalist capabilities of LLMs is considered to be one of the most relevant open problems in the field. The most recommended approach today is to use as many different benchmarks as possible, validating that models do not fail dramatically at any of those.

In the field of healthcare, LLMs are most commonly benchmarked using a few multiple-choice question-answering (MCQA) datasets. The main argument in favor of MCQA is that it provides exact matching metrics (e.g., accuracy from “Answer with A, B, C or D.") that can be reliably compared and easily interpreted by humans. But this same simplicity also entails a weakness, as the probability of producing one single output token (‘A’, ‘B’, ‘C’ or ‘D’) is hardly representative of the free discourse capacity needed to perform well at a broad range of natural language medical tasks (e.g., document summarization). At the same time, certain MCQA datasets (i.e., MedQA, PubMedQA, MedMCQA, HeadQA, MMLU, etc.) have been repeatedly used in the literature for model selection purposes^4^, showing signs of saturation and overfitting. While the evaluation used in this work goes well beyond MCQA, it also uses MCQA. To provide additional reassurances regarding the risk of contamination in this kind of testing, a more recent benchmark based on medical exams is also added (CareQA^17^, as recent as 2024). The multiple-choice question-answering evaluation results are presented in Section “MCQA Benchmarking”.

Open ended (OE) evaluation is complementary to MCQA. In OE, responses are of arbitrary length and structure. While this is a more realistic setup for the model to operate, the definition of a correct answer and its measurement becomes uncertain. Some measures (i.e., ROUGE, BLEU) are based on n-grams^20,21^, tracking the overlap of consecutive sets of words with those found in a reference answer (one of many possible ones). Others rate open answers by means of word perplexity, a metric borrowed from information theory which measures how likely it is to sample the correct answer from the model distribution, i.e., the inverse geometric mean of word-likelihoods for the ground truth under the model distribution^22^. Recent works have shown both approaches are uncorrelated^17^, measuring different aspects of answer quality. For this reason, this work observes both in Section “Open-ended Benchmarking” and draws limited conclusions from their values. The flexibility of OE also enables the integration of a variety of tasks, from clinical note-taking to document summarization, covering a broader set of clinical reasoning skills and applications.

The third type of evaluation considered in this work is based on human experts. This kind of evaluation provides relevant insights, particularly when considering the potential use of healthcare LLMs as assistants and decision-support mechanisms for humans. The limitations in human evaluation lie in the constrained scalability of a human-based study, and the existence of individual and collective biases for what constitutes a *good answer* (i.e., humans are known to prefer longer explanations^23^). Using a population of experts is necessarily limited in size and variety, yielding biases in the assessment towards the preferences of a specific population. Nonetheless, human evaluation provides a strong signal for user adoption, which is why a significant effort is conducted in this work, as presented in Section Human Evaluation.

To conclude the evaluation of the Aloe Family, and considering the relevance of model safety in healthcare, a model assessment is implemented in Section “Safety Evaluation”. This includes a study on how resilient models are to producing toxic or dangerous content even in the presence of malicious prompts designed to elicit such undesirable behaviors from LLMs.

These four evaluation methods (MCQA, OE, Human and Safety) are conducted on the four models from Aloe Family, and also on their corresponding baselines (i.e., the instruct version trained by the authors of the base model used by Aloe Beta). In some cases, one additional model is also used as a reference, Med42B, since this is similar to Aloe (healthcare fine-tune); it includes both small and big sizes and has its technical details reported.

### MCQA benchmarking

MCQA provides a quantifiable measure of performance for a well define task, through human interpretable meatrics such as precision and recall. This is feasible under a closed-format evaluation, and especially useful for model selection purposes, where hyperparameters are being selected.

Results of Table 2 indicate the Aloe Family models achieve top-level performance in all evaluated MCQA benchmarks. The Aloe models based on Llama 3.1 achieve moderate improvements with respect to its instruct version counterpart (less than 1 point *w.r.t*.Llama 3.1 Instruct), while the Aloe models based on Qwen 2.5 provide a significant boost when compared with the corresponding Qwen instruct model (+3.4 in accuracy on the 7B, +2.2 on the 72B) outperforming as a result all the Llama variants. This may be caused by a particularly thorough instruct tuning version on Llama 3.1 Instruct, with the potential presence of healthcare specific data. Overall, *Qwen2.5-Aloe-Beta-72B* is shown as the highest performing open model examined inside and outside of the Aloe Family.

**Table 2:** Results for MCQA medical benchmarks (accuracy, higher is better)

	Avg.	MultiMedQA	MedMCQA	MedQA	MMLU	CareQA
	*Open models - Small*					
Meta-Llama-3.1-8B-Inst.	67.15	63.79 ± 1.11	59.22 ± 1.49	63.71 ± 2.64	75.72 ± 2.71	69.95 ± 1.20
Llama3-Med42-8B	66.53	64.10 ± 1.11	60.20 ± 1.48	62.53 ± 2.66	75.08 ± 2.72	68.30 ± 1.22
*Llama3.1-Aloe-Beta-8B*	67.87	64.51 ± 1.10	59.57 ± 1.49	64.65 ± 2.63	76.50 ± 2.70	70.77 ± 1.19
Qwen2.5-7B-Inst.	66.96	61.66 ± 1.12	56.18 ± 1.50	61.59 ± 2.67	77.92 ± 2.58	72.14 ± 1.17
*Qwen2.5-Aloe-Beta-7B*	**70.38**	**66.39** ± **1.09**	**62.25** ± **1.47**	**65.36** ± **2.61**	**79.36** ± **2.51**	**74.56** ± **1.14**
	*Open models - Large*					
Meta-Llama-3.1-70B-Inst.	80.76	76.41 ± 0.98	72.15 ± 1.36	79.73 ± 2.21	87.45 ± 2.01	83.72 ± 0.97
Llama3-Med42-70B	80.06	76.28 ± 0.99	72.48 ± 1.35	78.16 ± 2.27	86.79 ± 2.09	82.80 ± 0.99
*Llama3.1-Aloe-Beta-70B*	80.88	76.54 ± 0.98	72.15 ± 1.36	79.73 ± 2.21	88.44 ± 1.92	83.19 ± 0.98
Qwen2.5-72B-Inst.	80.34	74.42 ± 1.00	69.26 ± 1.40	77.85 ± 2.28	88.81 ± 1.98	85.45 ± 0.92
*Qwen2.5-Aloe-Beta-72B*	$\underline{{\bf{82.54}}}$	$\underline{{\bf{77.64}}\pm {\bf{0.96}}}$	$\underline{{\bf{73.49}}\pm {\bf{1.34}}}$	**80.68** ± **2.17**	$\underline{{\bf{89.20}}\pm {\bf{1.95}}}$	$\underline{{\bf{86.78}}\pm {\bf{0.89}}}$
	*Closed models*					
MedPalm-2	–	76.04	71.30	79.70	**87.77**	–
GPT-4	–	**76.59**	**72.40**	$\underline{{\bf{81.40}}}$	87.37	–
	*Aloe Model + In-Context Learning*					
*Llama3.1-Aloe-Beta-8B*	77.50	75.08	70.76	80.60	83.20	75.45
*Qwen2.5-Aloe-Beta-7B*	76.85	74.34	70.93	76.12	83.65	76.71
*Llama3.1-Aloe-Beta-70B*	84.82	79.99	75.14	**86.88**	90.58	86.67
*Qwen2.5-Aloe-Beta-72B*	$\underline{{\bf{85.68}}}$	**81.35**	**77.77**	85.94	**90.89**	$\underline{{\bf{88.13}}}$
	*Closed Models + In-Context Learning*					
MedPalm-2 (choose best)	–	78.30	72.3	86.5	89.9	–
GPT-4 (Medprompt)	–	$\underline{{\bf{83.66}}}$	$\underline{{\bf{79.1}}}$	$\underline{{\bf{90.2}}}$	$\underline{{\bf{94.25}}}$	–

The first block reports 0 shot results, with models sorted by size. Second block shows models boosted by in-context learning methods. For Aloe this is with SFR-Embedding-Mistral, 20 ensembles, and 5 few-shots examples. In bold best in the model size range. Underlined and bold best overall. Closed model results are not reproduced. These are reported by the authors of Medprompt^1^ and MedPalm-2^2^.

The bottom part of Table 2 shows performance results on the Aloe models when integrating an in-context learning pipeline based on RAG (as described in Section In-Context Learning).

### Medical fields

In this section, we review the same MCQA evaluation, with results separated by medical field. This is done first to assess how reliable LLM performance is across healthcare categories and second to provide a model selection guide to potential users who may be interested in one particular field. The same questions reported in Table 2 are reported in Table 3. These are classified into pre-defined medical categories by the Llama-3-70B-Instruct model, using a tool developed for this task, which includes a prompt to that end (*e.g*., *... you will be given a medical question. Your job is to categorize the question into one of the following categories*...). To list of categories used was first extracted as an exhaustive list of 61 different labels, belonging to medicine, nursing and dentistry specialities. These 61 were reduced to simplify the classifier task, grouping those that were found to be overlapping and removing those that did not have enough samples after classification. As a result, 17 categories were selected (see Table 3). Classifier performance was assessed through human supervision of random samples, as ground truth labels were not available.

**Table 3:** Model accuracy at MCQA by medical specialty

Model	Cardiology	Hematology	Respiratory	Urology	Orthopedics	Surgery
Meta-Llama-3.1-8B-Inst.	0.65	0.69	0.61	0.65	0.61	0.58
Llama3-Med42-8B	0.65	0.70	0.65	0.66	0.56	0.58
*Llama3.1-Aloe-Beta-8B*	0.66	0.67	0.66	0.71	0.63	0.56
Qwen2.5-7B-Inst.	0.61	0.71	0.66	0.62	0.65	0.56
*Qwen2.5-Aloe-Beta-7B*	0.67	0.76	0.70	0.72	0.69	0.69
Meta-Llama-3.1-70B-Inst.	0.78	0.82	0.81	0.76	0.75	0.69
Llama3-Med42-70B	0.78	0.81	0.75	0.77	**0.78**	**0.74**
*Llama3.1-Aloe-Beta-70B*	0.79	0.83	0.81	0.75	0.74	0.69
Qwen2.5-72B-Inst.	0.76	0.82	0.81	**0.83**	0.75	0.72
*Qwen2.5-Aloe-Beta-72B*	**0.80**	**0.85**	**0.84**	0.81	**0.78**	0.71

Model	Neurology	Nephrology	Gastroenterology	Obstetrics	Gynecology	Allergy
Meta-Llama-3.1-8B-Inst.	0.66	0.70	0.68	0.59	0.74	0.81
Llama3-Med42-8B	0.65	0.69	0.70	0.59	0.70	0.78
*Llama3.1-Aloe-Beta-8B*	0.67	0.70	0.68	0.57	0.75	0.79
Qwen2.5-7B-Inst.	0.66	0.67	0.63	0.60	0.70	0.79
*Qwen2.5-Aloe-Beta-7B*	0.71	0.70	0.65	0.76	0.84	0.77
Meta-Llama-3.1-70B-Inst.	0.80	0.84	0.80	0.75	0.84	**0.92**
Llama3-Med42-70B	0.82	0.84	0.80	0.78	0.82	0.88
*Llama3.1-Aloe-Beta-70B*	0.80	0.84	0.81	0.76	0.84	**0.92**
Qwen2.5-72B-Inst.	0.82	0.84	0.75	0.73	**0.87**	0.88
*Qwen2.5-Aloe-Beta-72B*	**0.83**	**0.88**	**0.82**	**0.80**	0.85	**0.92**

Model	Dermatology	Endocrinology	Rheumatology	Ophthalmology	Oncology
Meta-Llama-3.1-8B-Inst.	0.70	0.69	0.71	0.64	0.76
Llama3-Med42-8B	0.72	0.70	0.68	0.68	0.74
*Llama3.1-Aloe-Beta-8B*	0.73	0.72	0.71	0.62	0.76
Qwen2.5-7B-Inst.	0.69	0.72	0.72	0.67	0.73
*Qwen2.5-Aloe-Beta-7B*	0.73	0.77	0.73	0.68	0.86
Meta-Llama-3.1-70B-Inst.	0.82	0.84	0.85	**0.86**	0.86
Llama3-Med42-70B	0.82	0.83	0.85	0.83	0.87
*Llama3.1-Aloe-Beta-70B*	0.82	0.83	**0.89**	0.85	0.86
Qwen2.5-72B-Inst.	0.83	0.84	0.86	0.78	0.83
*Qwen2.5-Aloe-Beta-72B*	**0.87**	**0.85**	**0.89**	0.82	**0.89**

In bold, best model for each field.

The performance of LLMs according to Table 3 shows significant variance, with some fields being more challenging (*e.g*., Surgery, Orthopedics) than others (e.g., Allergy, Oncology). The *Aloe-Beta-72B* model achieves top performance in 13 out of 17 categories, although in most cases, two or more models are close in top performance. These results can be used as a guidance for model selection for field-specific applications.

### Open-ended benchmarking

Open-ended tasks evaluate text fluency, overall coherence, and the model’s ability to produce consistent outputs (something not captured by MCQA tests). In the context of evaluating healthcare LLMs, assessing their performance across a broad spectrum of tasks is essential to ensure that these models can address the complex requirements of clinical settings. Such is the motivation of including an open-ended (OE) evaluation. This subsection focuses on evaluating tasks that require generating context-specific outputs instead of selecting answers from a predefined set of limited options (*i.e*., MCQA). Notice the OE process remains an ongoing effort^17,24,25^ since there is no standardized set of benchmarks that tests all the medical and clinical applications and tasks. Therefore, the benchmarks used for OE evaluations are extensive but not exhaustive. In detail, the set of tasks included are derived from the suite presented in ref. ^17^.

- **Clinical note-taking**: Generating notes based on conversations between healthcare providers and patients or other clinical interactions, using MTS-Dialog^26^ and ACI-Bench^27^.
- **Diagnosis and treatment recommendations**: Providing clinical guidance based on a patient’s condition, evaluated with the MedText dataset.
- **Medical classification**: Categorizing medical texts into specific categories, using two benchmarks: Medical Text for Classification^28^ and Medical Transcriptions.
- **Medical factuality**: Measuring the factual accuracy of medical responses, evaluated with the OLAPH dataset^29^.
- **Open-ended medical questions**: Responding to complex medical queries, with performance measured using CareQA Open, MedDialog Raw^30^, and MEDIQA2019^31^.
- **Question entailment**: Determining whether one question logically follows from another, using MedDialog Qsumm^30^.
- **Relation extraction**: Identifying and extracting relationships between entity pairs (*e.g*., gene/protein, disease, chemical), evaluated using the BioRED dataset^32^.
- **Summarization**: Condensing medical information into concise and coherent summaries, using the MIMIC-III dataset^33^.

Results for all OE tasks are reported in Table 4 (n-gram metrics) and 5 (perplexity metrics). The former results are inconsistent, showing a high variance across models. *All* reported models are highly competitive in one or more benchmarks. Remarkably, small models (7B and 8B) are close in performance to large models and, at times, even outperform them (e.g., MTS-Dialog, MEDIQA2019). All in all, these results indicate the Aloe training strategy has not significantly modified the model’s capacity to produce coherent dialogue and remains competitive on all tasks considered. These highly variable results are also influenced by the used metrics (ROUGE1), which measure the overlap between generated and reference texts. Similar results were observed with ROUGE2 and ROUGEL, which measure bigram and longest-gram overlap, respectively. While ROUGE1 is an efficient metric for assessing the relevance and coverage of generated content, it focuses on lexical similarity without accounting for semantic meaning or coherence.

**Table 4:** Results across different tasks

	Clinical-note taking		Diagnosis and treatment recommendation	Medical classification		Medical factuality
	ACI Bench	MTS-Dialog	MedText	Medical text classification	Medical transcriptions	OLAPH
	ROUGE1 *↑*	ROUGE1 *↑*	ROUGE1 *↑*	Accuracy *↑*	Accuracy *↑*	ROUGE1 *↑*
Meta-Llama-3.1-8B-Inst.	15.85	6.88	**26.45**	35.14	33.61	**30.14**
Llama3-Med42-8B	**21.74**	**10.24**	26.44	**53.50**	**36.67**	26.86
*Llama3.1-Aloe-Beta-8B*	20.38	7.61	25.51	48.51	33.03	27.05
Qwen2.5-7B-Inst.	17.90	$\underline{{\bf{10.31}}}$	27.32	63.48	**38.27**	**28.79**
*Qwen2.5-Aloe-Beta-7B*	**20.34**	5.52	**28.77**	**63.89**	35.89	24.46
Meta-Llama-3.1-70B-Inst.	20.41	11.02	**28.72**	62.23	38.01	32.52
Llama3-Med42-70B	**21.23**	4.97	25.01	$\underline{{\bf{64.59}}}$	**38.57**	23.17
*Llama3.1-Aloe-Beta-70B*	12.34	**11.51**	28.34	59.94	38.17	**31.83**
Qwen2.5-72B-Inst.	17.31	3.95	31.62	**63.41**	$\underline{{\bf{39.29}}}$	$\underline{{\bf{33.21}}}$
*Qwen2.5-Aloe-Beta-72B*	$\underline{{\bf{26.86}}}$	**9.19**	$\underline{{\bf{32.81}}}$	57.66	35.43	28.92

	Open-ended medical questions			Question entailment	Relation extraction	Summarization
	CareQA (open)	MedDialog Raw	MEDIQA2019	MedDialog Qsumm	BioRED	MIMIC-III
	ROUGE1 *↑*	ROUGE1 *↑*	ROUGE1 *↑*	ROUGE1 *↑*	Accuracy *↑*	ROUGE1 *↑*
Meta-Llama-3.1-8B-Inst.	**14.94**	**12.74**	15.38	**14.91**	46.29	10.40
Llama3-Med42-8B	**14.94**	12.06	16.63	13.31	**54.12**	**14.30**
*Llama3.1-Aloe-Beta-8B*	14.36	12.06	**18.61**	14.45	49.75	9.84
Qwen2.5-7B-Inst.	**15.73**	11.77	19.29	**16.17**	**54.32**	**15.53**
*Qwen2.5-Aloe-Beta-7B*	12.92	**12.01**	$\underline{{\bf{19.37}}}$	10.94	44.86	12.70
Meta-Llama-3.1-70B-Inst.	$\underline{{\bf{17.85}}}$	13.04	**18.72**	$\underline{{\bf{16.45}}}$	$\underline{{\bf{63.28}}}$	12.62
Llama3-Med42-70B	12.66	11.57	14.42	12.65	51.58	11.66
*Llama3.1-Aloe-Beta-70B*	17.61	$\underline{{\bf{12.91}}}$	**18.72**	15.61	59.92	**14.81**
Qwen2.5-72B-Inst.	**17.26**	**12.04**	**18.55**	**16.39**	56.26	$\underline{{\bf{16.87}}}$
*Qwen2.5-Aloe-Beta-72B*	15.41	11.41	18.44	13.54	**58.39**	14.27

The first row of the header indicates the task, the second specifies the benchmark, and the third shows the metric used (ROUGE1 or accuracy). The rows are grouped into four blocks: (1) Llama 3.1-8B instruct and its fine-tunes (Med42 and Aloe-Beta), (2) Qwen 2.5-7B instruct and its Aloe-Beta fine-tune and the corresponding larger models in (3) and (4). Bold values indicate the best results within the block, while bold and underlined values represent the overall best.

Perplexity results, which measure the predictive strength of the models, are slightly more consistent. In Table 5, larger models generally outperform smaller ones, and fine-tuned configurations (e.g., Med42 and Aloe) improve performance over their corresponding baselines, underscoring the importance of task-specific training. According to this metric, and in agreement with the results in MCQA evaluation, *Qwen2.5-Aloe-Beta-72B* is the best performing open model examined inside and outside of the Aloe Family.

**Table 5:** Perplexity scores across different tasks and benchmarks for various models (lower is better)

	Clinical-note taking		Open-ended medical questions			Medical factuality
	ACI Bench	MTS-Dialog	CareQA (open)	MedDialog Raw	MEDIQA2019	OLAPH
	Perplexity *↓*					
Meta-Llama-3.1-8B-Instruct	14.19	**120.13**	573.72	80.16	6.18	6.93
Llama3-Med42-8B	**8.42**	141.88	**445.40**	76.77	5.74	6.70
*Llama3.1-Aloe-Beta-8B*	13.94	138.35	495.84	**76.06**	**5.54**	**6.53**
Qwen2.5-7B-Instruct	15.25	188.60	**1733.92**	98.12	5.24	7.63
*Qwen2.5-Aloe-Beta-7B*	**13.35**	**134.25**	1998.95	**74.86**	**4.8**	**7.45**
Meta-Llama-3.1-70B-Instruct	11.44	**93.94**	406.88	**60.91**	2.89	**6.11**
Llama3-Med42-70B	$\underline{{\bf{7.80}}}$	94.85	$\underline{{\bf{354.56}}}$	62.39	**2.54**	7.19
*Llama3.1-Aloe-Beta-70B*	12.69	116.67	522.78	70.15	2.94	6.37
Qwen2.5-72B-Instruct	15.12	101.52	551.91	66.40	2.12	6.34
*Qwen2.5-Aloe-Beta-72B*	**10.15**	$\underline{{\bf{90.08}}}$	**435.19**	$\underline{{\bf{54.11}}}$	$\underline{{\bf{1.90}}}$	$\underline{{\bf{5.93}}}$

The table shows the performance of Llama3.1 and Qwen2.5 along with their fine-tuned versions. Bold values indicate the best performance within each task, while bold and underlined values highlight the overall best results.

### Human evaluation

Expert preferences assess the potential clinical usefulness of models, their safety, and the degree to which alignment and DPO produce outputs preferred by human experts. In addition, these evaluations can help identify shortcomings that automated methods (like MCQA or OE) fail to capture.

**Fig. 3:**
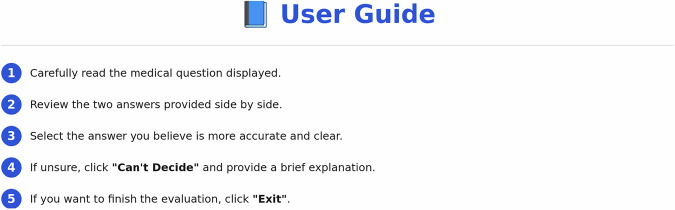
Guidelines given to medical experts for the human evaluation task. Screenshot of the instructions given to experts for evaluating model responses.

To assess the quality of the model’s responses according to human expertise, we conduct an evaluation with medical experts. These experts are gathered from a mailing list including 13,000 physicians who are officially registered within a medical college in Spain. Although participants are requested to have at least five years of clinical practice, we do not store such details (name, age, speciality or years of experience) to preserve the privacy of participants and to avoid any personal data in adherence to European privacy regulations (GDPR). The task is defined below (see Fig. 3), and designed to reduce the relevance of such personal data (e.g., questions are related with general medicine, and articulated by patients, not experts). Notice the population of experts shares the same geographical location, and is likely to include any bias found in that distribution.

Given the cost in medical expert hours this evaluation is limited to comparing the larger models in the Aloe Family with the official Llama 3.1 and Qwen 2.5 instruct versions. The study is designed as a binary choice in which humans evaluate pairs of models. After being presented with a question, and two possible answers for it (produced by two hidden models) the evaluator must specify which one they prefer.

The questions used in this evaluation were gathered from Reddit, specifically the “*HealthAdvice*” subreddit (accessed on November 6, 2024). This choice was made for three main reasons. First, the answers to these questions are not highly specialized, meaning that most doctors could evaluate the quality of answers regardless of their medical field of expertisee. Second, it represents a real use case: people currently seek medical advice on Reddit, and in the future, they may turn to LLMs for similar guidance; the way in which questions are written is representative of what LLMs may face in the real world. And third and last reason, such recent data is unlikely to be included in existing training datasets (e.g., it lacks a structured ground truth), reducing the chances of data contamination.

After anonymizing questions to delete the presence of personal data, a total of 669 questions were collected. Four models were used to answer all these questions: *Aloe-Beta-70B*, *Aloe-Beta-72B*, Llama 3.1-70B, and Qwen 2.5-72B. The same questions are independently presented to evaluators in fixed order, each question being shown only once to each evaluator, together with a single response pair. Since there are six possible response pairs to every question (*Aloe-Beta-70B* vs *Aloe-Beta-72B*, *Aloe-Beta-70B* vs Llama3.1-70B, *Aloe-Beta-72B* vs Qwen2.5-72B, *etc*.), the pair of answers seen by different evaluators on the same question may vary. This design prevents evaluators from performing redundant efforts which would affect adherence, and increases the consistency of results by reducing variance among questions. However, it also reduces the possibility of doing inter-evaluator agreement studies.

A total of 49 evaluators participated in this study, producing a total of 695 unique responses (preferences). Evaluators were selected based on medical background and professional experience. To reduce language and interpretation biases, only evaluators with intermediate to advanced English proficiency were included. To address expertise differences, multiple evaluators assessed each question, minimizing the impact of outlier opinions. Finally, to reduce fatigue bias, a progress bar tracked progress. The interface also allowed evaluators to resume the evaluation on a different day. Due to the limited number of evaluators, results are possibly affected by other biases (*e.g*., demographics).

**Fig. 4:**
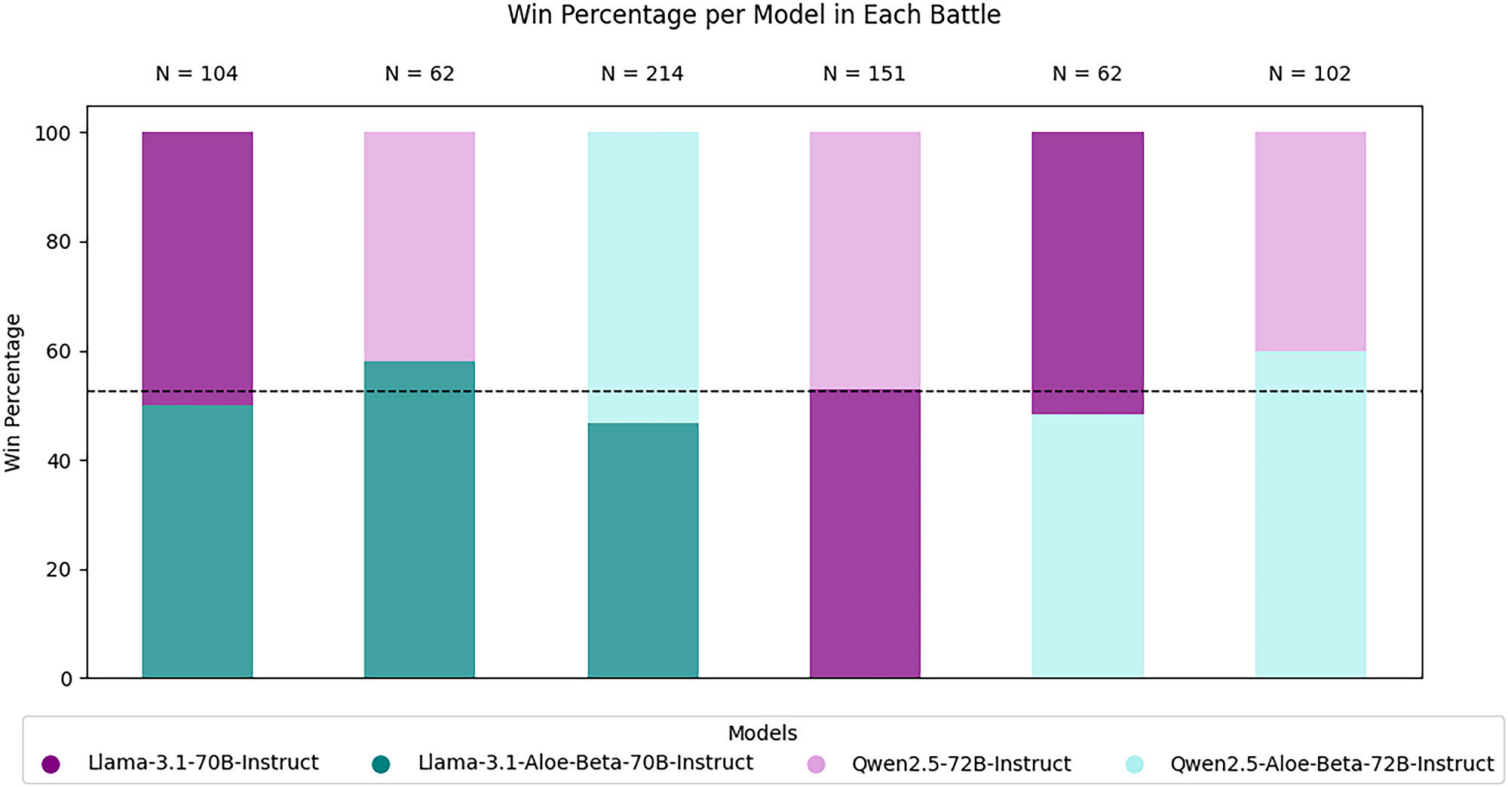
Pairwise, model preference of healthcare experts on medical questions from Reddit. N values indicate number of responses for that particular pair of models.

Figure 4 shows a summary of results. For every pair of models evaluated, the plot shows which one was preferred when both were presented to expert evaluators. In general, choices are balanced. The comparison between the two Aloe models (third bar) shows both models are similarly preferred by medical doctors. After computing binomial tests to assess statistical significance, no battle showed statistically significant scores (*p* < 0.05). These result highlight that, due to their nature, the medical questions gathered are easy to answer for the models following healthcare professional criteria. The choice of one model over the other is relegated to the personal preference of the doctors, with limited statistical relevance.

### Safety evaluation

Any model released to the public (general or specialized), needs to be tested for their resilience to malicious inputs, and their refusal to follow dangerous instructions. In particular, safety assessment measures the resilience of models to produce unsafe responses in the presence of adversarial prompts. That is, when fed inputs explicitly designed to elicit dangerous outputs (i.e., jailbreaking). This is particularly relevant for LLMs in the healthcare domain, considering the critical nature of their application, together with the high level of reliability presumed from such systems. The Aloe Family models include specific training to increase their safety. The data used for that end is described in Section Jailbreaking, and the learning process itself in Section Model Alignment.

To assess the safety of models, an independent benchmark is used (S-Eval^34^) which includes ten attack styles (Chain of Utterances, Code Injection, Compositional Instructions, Deep Inception, Goal Hijacking, In-Context Attack, Instruction Encryption, Instruction Jailbreak, Positive Induction, and Reverse Induction), and eight topic categories (Inappropriate Suggestions, Physical and Mental Health, Crimes and Illegal Activities, Cybersecurity, Data Privacy, Ethics and Morality, Extremism, and Hate Speech). Safety is measured as the fraction of those prompts which successfully produce an unsafe response from the model, that is, attack success rate (ASR, lower is better). Safety of responses is evaluated with *Llama Guard 3 8B*, which has been shown to align strongly with human safety preferences^35^.

Table 6 summarizes results separated by attack style. On average, the two biggest Aloe models are the safest in this experimentation, showing remarkably high resistance to all attack styles. Both *Llama3.1-Aloe-Beta-70B* and *Qwen2.5-Aloe-Beta-72B* have attack success rate is below 9%. This represents significant gains *w.r.t*.their respective baseline. In contrast, smaller models are more sensitive to jailbreaking. The *Qwen2.5-Aloe-Beta-7B* slightly improves its instruct counterpart (Qwen 2.5 7B Instruct), while the *Llama3.1-Aloe-Beta-8B* does not. Table 7 shows results from the same experimentation, but separated by safety topic. Results are quite similar in this case, with bigger models getting the most gain *w.r.t*.their baseline. Over-refusal rates were evaluated separately^35^, and found to be minimal. Overall, these results indicate the effectiveness of the safety training implemented, particularly for large models, and motivate the use of larger LLMs in critical environments.

**Table 6:** Attack success rates (ASR, lower is better) across 10 different jailbreaking attacks from the S-Eval safety benchmark

	Avg.	S-Eval									
		CoU	CInj	CIns	DI	GH	ICA	InsE	InsJ	PInd	RInd
	Attack Success Rate *↓*										
Meta-Llama-3.1-8B-Inst.	**0.17**	**0.38**	0.29	**0.17**	0.18	$\underline{{\bf{0.03}}}$	**0.04**	**0.03**	0.42	**0.09**	0.07
Llama3-Med42-8B	0.20	0.73	$\underline{{\bf{0.16}}}$	0.19	**0.14**	0.05	0.08	0.04	0.42	0.15	$\underline{{\bf{0.04}}}$
*Llama3.1-Aloe-Beta-8B*	0.22	0.63	0.30	0.24	0.19	0.06	0.07	**0.03**	0.53	0.12	$\underline{{\bf{0.04}}}$
Qwen2.5-7B-Inst.	0.29	0.83	0.42	0.31	0.25	**0.12**	**0.20**	0.03	0.50	**0.26**	**0.06**
*Qwen2.5-Aloe-Beta-7B*	**0.27**	**0.68**	**0.22**	**0.25**	**0.09**	0.19	0.36	$\underline{{\bf{0.01}}}$	**0.48**	0.41	0.08
Meta-Llama-3.1-70B-Inst.	0.15	0.21	0.27	0.30	0.06	0.09	0.18	0.05	0.22	0.13	0.06
Llama3-Med42-70B	0.28	0.92	$\underline{{\bf{0.16}}}$	0.22	0.08	0.14	0.34	0.05	0.62	0.28	0.07
*Llama3.1-Aloe-Beta-70B*	$\underline{{\bf{0.07}}}$	$\underline{{\bf{0.00}}}$	0.24	0.14	**0.03**	**0.05**	**0.06**	0.04	$\underline{{\bf{0.08}}}$	**0.07**	0.06
Qwen2.5-72B-Inst.	0.14	0.01	0.38	0.28	0.08	0.06	0.08	0.09	0.24	0.13	0.05
*Qwen2.5-Aloe-Beta-72B*	**0.08**	$\underline{{\bf{0.00}}}$	**0.21**	**0.22**	$\underline{{\bf{0.01}}}$	**0.04**	$\underline{{\bf{0.03}}}$	**0.07**	**0.18**	$\underline{{\bf{0.06}}}$	$\underline{{\bf{0.04}}}$

The table shows the performance of the Llama 3.1 and Qwen 2.5 instruct models, along with fine-tuned medical models and the Aloe Family (in italics). Bold values indicate the best performance across models from the same base, bold and underlined refers to the overall best on each attack style. Attacks are Chain of Utterances (CoU), Code Injection (CInj), Compositional Instructions (CIns), Deep Inception (DI), Goal Hijacking (GH), In-Context Attack (ICA), Instruction Encryption (InsE), Instruction Jailbreak (InsJ), Positive Induction (PInd), and Reverse Induction(RInd).

**Table 7:** Attack success rates (ASR, lower is better) across the 8 risk categories from the S-Eval safety benchmark

	Avg.	S-Eval							
		IS*	PMH*	CIA	CS	DP	EM	Ext	HS
	Attack Success Rate *↓*								
Meta-Llama-3.1-8B-Inst.	**0.16**	0.28	**0.13**	**0.20**	**0.20**	**0.11**	**0.15**	**0.15**	**0.13**
Llama3-Med42-8B	0.19	**0.27**	**0.13**	0.24	0.27	**0.11**	0.17	0.21	0.17
*Llama3.1-Aloe-Beta-8B*	0.21	0.32	0.15	0.27	0.34	**0.11**	0.18	0.22	0.16
Qwen2.5-7B-Inst.	0.29	0.35	0.20	0.37	**0.45**	0.14	0.25	**0.31**	0.26
*Qwen2.5-Aloe-Beta-7B*	**0.27**	**0.28**	**0.16**	**0.36**	0.46	**0.10**	**0.23**	0.33	**0.25**
Meta-Llama-3.1-70B-Inst.	0.15	0.25	0.09	0.19	0.20	0.09	0.12	0.17	0.13
Llama3-Med42-70B	0.28	0.34	0.19	0.36	0.41	0.14	0.25	0.32	0.26
*Llama3.1-Aloe-Beta-70B*	$\underline{{\bf{0.07}}}$	$\underline{{\bf{0.16}}}$	$\underline{{\bf{0.04}}}$	$\underline{{\bf{0.10}}}$	$\underline{{\bf{0.09}}}$	$\underline{{\bf{0.04}}}$	**0.06**	**0.07**	$\underline{{\bf{0.05}}}$
Qwen2.5-72B-Inst.	0.13	0.24	0.08	0.17	0.22	0.07	0.10	0.12	0.10
*Qwen2.5-Aloe-Beta-72B*	**0.08**	**0.18**	**0.05**	**0.11**	**0.12**	$\underline{{\bf{0.04}}}$	$\underline{{\bf{0.05}}}$	$\underline{{\bf{0.06}}}$	**0.06**

The table shows the performance of the Llama 3.1 and Qwen 2.5 instruct models, along with fine-tuned medical models and the Aloe Family (in italics). Bold values indicate the best performance across models from the same base, bold and underlined refers to the overall best on each attack style. Topics include Inappropriate Suggestions (IS), Physical and Mental Health (PMH), Crimes and Illegal Activities (CIA), Cybersecurity (CS), Data Privacy (DP), Ethics and Morality (EM), Extremism (Ext), and Hate Speech (HS). The topics with an asterisk (*), “Inaproppriate Suggestions” and “Physical and Mental Health”, include or are entirely composed of healthcare-related prompts.

### Risk assessment

The release of an LLM designed for use in the medical field motivates an assessment of potential risks and mitigation strategies. Beyond the post-training efforts done in Aloe to increase safety, other technical mechanisms exist for further reassurances. Watermarking, while promising, is currently ineffective in text outputs. Guardrails, while highly effective, represent an independent layer of safety wrapped around an LLM, pattern matching its inputs and outputs as a black box.

To identify which additional mitigation tools are applicable, first we need to recognize the potential dangers related to the publication of the Aloe Family. For that, we follow the six points proposed in ref. ^36^, describing three main risks specific to the healthcare domain. General purpose risks assessments of LLMs are numerous in existing literature, and typically provided by the author of the pre-trained model.

### Risk 1: Healthcare professional impersonation

Summary: Impersonating medical experts is a fraudulent behavior which currently generates billions of dollars in profit. A model such as Aloe could increase the efficacy of such deceiving activities, making them more widespread. The main preventive actions are public education on the unreliability of digitized information and the importance of medical registration and legislation enforcing AI-generated content disclaimers.

1. Threat Identification: Healthcare professional impersonation
  Execution: Using Aloe, one can produce plausible medical text, hide its synthetic origin, and use it to impersonate a medical expert to manipulate others.
  Malicious actors: Individuals seeking economic gains by getting others to pay them as medical experts. Actors with a specific interest in someone’s medical care.
  Resources: A certain amount of initial trust or visibility would be needed (e.g., a fake clinic webpage). If the impersonation targets a specific individual, knowledge of their past condition would be necessary. Since interactions are eventually likely to happen in real time, a high throughput inference set-up for the LLM would be needed.
2. Existing risk: The impersonation of medical experts is an illegal activity already being conducted. People are practising as medical experts without the proper training all over the world, generating millions of dollars and endangering public health.
3. Existing defences: The main mechanism against impersonation is proper identification and certification. These are typically implemented by the College of Physicians or Medical Associations, which issue official documentation and recognize its members. This goes hand in hand with public literacy, which emphasizes the importance of relying only on certified professionals.
4. Marginal risk: A healthcare LLM increases this risk by facilitating the impersonation on digital means of communication (e.g., chats with doctors). This family of models increases the risk in all non-face-to-face interactions.
5. New defences: Model-level guardrails on deployed versions of Aloe, integrating classifiers to detect and refuse impersonation prompts. and trigger refusals or redirections. Other methods like user authentication and rate limiting, to prevent large-scale automated campaigns. This could be expanded to implementing behavioral authentication in healthcare related contexts, to guarantee there is a human on the other side of the communication channel. Public literacy on the increasing unreliability of digital content’s true origin and nature. Prioritization of face-to-face interactions for critical issues such as healthcare treatment. Public legislation enforcing the addition of disclaimers on all AI-generated content. Regarding this last point, integration with AI Output Watermarking would be desirable.
6. Uncertainty and assumptions: This assessment assumes risk is limited to digital interactions and constrained by inference latency. Improvements in inference speed, and the integration of models enabling other modalities (*e.g*., voice to text, text to voice) may export the risk to other settings.

### Risk 2: Medical decision-making without professional supervision

Summary: While this is already an issue in modern societies (e.g., self-medication) a model such as Aloe, capable of producing high-quality conversational data, can facilitate self-delusion, particularly in the presence of sycophancy. By producing tailored responses, it can also be used to generate actionable answers. Public literacy on the dangers of self-diagnosis is one of the main defences, together with the introduction of disclaimers and warnings on the models’ outputs.

1. Threat Identification: Medical decision-making without professional supervision
  Execution: An individual decides to obtain a diagnosis, plan treatment, or conduct other complex medical decision-making through a healthcare LLM without proper supervision. Such an individual follows the advice of such a model without ever consulting with a medical expert.
  Malicious actors: Anyone without sufficient knowledge of the limitations of healthcare LLMs.
  Resources: Access to a healthcare LLM for inference without supervision.
2. Existing risk: Self-diagnose and self-medication is already an issue in most countries. Many individuals are willing to disregard professional advice and follow information from other sources (, Internet, social media).
3. Existing defences: Medications are highly controlled substances. Diagnostic tools are only accessible to trained professionals. Public announcements regarding obtaining professional advice are regularly made in most countries.
4. Marginal risk: The quality of LLM outputs can encourage individuals to overestimate the reliability and factuality of the information provided, increasing the number of people vulnerable to this risk. The personalization of LLMs responses to user queries can also become more actionable.
5. New defences: Model-level guardrails based on classifiers detecting self-diagnosis or self-medication requests from users, driving the model towards responses that the referral to professionals. This includes the restriction of dose calculations and prescription, as well as adding warnings and disclaimers when answering specific medical questions. Public literacy on the limitations in the factuality of LLMs, particularly when lacking human supervision, illustrated with hallucination examples.
6. Uncertainty and assumptions: This risk assumes the availability of a medical expert to the general, which should always be favored before an AI-based model’s output. However, many world populations lack access to such expertise. For some of these, the alternative to a healthcare LLM may be no medical advice. In this setting, this risk needs to be reassessed.

### Risk 3: Access to information on dangerous substances or procedures

Summary: While the literature on sensitive content can already be found on different sources (*e.g*., libraries, internet, dark web), LLMs can centralize such access, making it nearly impossible to control the flow of such information. Model alignment can help in that regard, but the effects remain insufficient so far, as jailbreaking methods still overcome it.

1. Threat Identification: Accessing information on dangerous substances or procedures
  Execution: Query the LLM to obtain information on controlled or dangerous substances or procedures, using such information to endanger human lives and public health.
  Malicious actors: An individual wanting to produce or acquire controlled or dangerous substances or to conduct a dangerous procedure.
  Resources: Access to a healthcare LLM for inference without supervision.
2. Existing risk: The information healthcare LLMs are trained with is publicly available to all. A skilled or motivated user can gather information regarding controlled or dangerous substances from traditional sources (e.g., library, wikipedia), as well as from opaque sources (e.g., dark web).
3. Existing defences: While information on controlled or dangerous substances is available, authors typically do not explicitly mention all the details needed to conduct illegal or harmful activities. There are also far more explicit sources (e.g., The Anarchist Cookbook) which are censored and prosecuted in certain jurisdictions with limited effectivity.
4. Marginal risk: A healthcare LLM provides simplified access to information on controlled or dangerous substances such as drugs, as well as critical medical procedures. The LLM’s ability to *digest* and format information facilitates the retrieval of such sensitive knowledge and makes it more accessible to the general public. Limiting access to models becomes even more complicated than limiting access to certain books, as models are digital artefacts (this is borderline since books became digitalized, too).
5. New defences: Performing alignment training (e.g., DPO) to prevent the LLM to discuss sensitive topics is a feasible approach, although its effectiveness is limited due to current jailbreaking methods. Safety classifiers could be added, focused on the recognition of harmful or dangerous procedures or substances, triggering a refusal to reply. Additionally, rate limiting methods can help detect systematic probing for sensitive information and system weaknesses. In extreme cases, access control can be implemented by restricting access to the sensitive data needed to train specific models, so that only licensed and registered users can use them.
6. Uncertainty and assumptions: Even if information on controlled or dangerous substances is available, we assume physical access to the components and necessary ingredients is far more complicated.

## Discussion

The development of LLMs for healthcare is a complex process that involves three main components, explored at depth in this work. That is *data* (selection, pre-processing and generation as seen in Section Training Data), *training* (domain adaptation an instruct tuning, model alignment and merging, as seen in Section Learning Stages) and *evaluation* (MCQA, by medical field, open-ended, human and safety, as seen in Section Results). By tackling all this topics, this work provides a comprehensive guide for developing medical LLMs, identifying strategies that have been found to robustly yield improvements across model sizes (7B, 8B, 70B, 72B) and families (Llama 3.1, Qwen 2.5). Our contributions extend beyond previous effort for healthcare LLMs, address practical deployment challenges, such as model merging techniques, jailbreaking protection mechanisms, RAG integration for knowledge enhancement, risk assessment frameworks, and human expert evaluation protocols. These components are essential for developing LLMs that are not only performant but also safe and reliable in clinical contexts.

To contribute to the open LLM community and enable full reproducibility, this work is released with model weights for the four Aloe Beta models, the curated training and evaluation datasets, and the complete RAG pipeline. Together, these components enable the Aloe models to achieve performance comparable to leading proprietary services (Fig. 2), while remaining fully accessible for the research community. The results obtained in the evaluation, show how the Aloe Family models are generalist healthcare models (see field-specific performance reported in Table 3), performing competitively in a variety of 19 medical tasks. This is shown through their consistent results on all evaluations conducted, and makes them candidates for further specialization through additional fine-tuning or RAG integration. The efficiency and limited computational footprint of the proposed training scheme enables this recipe to easily replicated and reused by the community.

Beyond the technical insights gained during this work, the extensive evaluation conducted produced other outcomes of value. To start with, the demonstration of how open healthcare models can compete with the best private alternatives when deployed on similar setups (i.e., with RAG on MCQA), which is something that had yet to be shown prior to this work. The study by medical field shows up to 20% drops in model performance, particularly when comparing specialities where physical interaction and external devices are often involved (i.e., Orthopedics or Surgery) with fields based on pure knowledge memorization (i.e., Allergy), demonstrating an uneven progress towards application. In contrast, open-ended evaluation shows inconclusive results, hampering their utility for model selection and motivating their use as simple sanity checks.

In the human evaluation, results indicate current LLMs are capable of providing reliable advice to primary healthcare questions, highlighting the maturity of the field. This seems to be a feature available in both healthcare models, like Aloe, and generalist models like Llama or Qwen, motivating the use of healthcare specialist models for harder, domain-expert applications. Finally, the safety evaluation illustrates the weakness of models, as well as the impact of applying relatively cheap alignment methods towards preventing potentially dangerous or harmful responses. These outcomes, and their potential for practical deployment, need to be contextualized within the risks that LLM entail in the field of healthcare, such as professional impersonation, decision-making without professional supervision, and access to potentially harmful information, as reviewed in the risk assessment included with this work.

While the conducted evaluation is broad within the context of the related work, it is not broad enough within the context of real world medicine. We acknowledge there are many other aspects of healthcare practice beyond the ones tackled here, that need to be addressed to guarantee a safe deployment of this technology. This include real-time device interpretation, procedural decision-making requiring imaging or device data, legal/regulatory workflows, longitudinal patient management, prospective clinical trials or clinician-in-the-loop evaluation for outcomes, among others.

## Methods

**Fig. 5:**
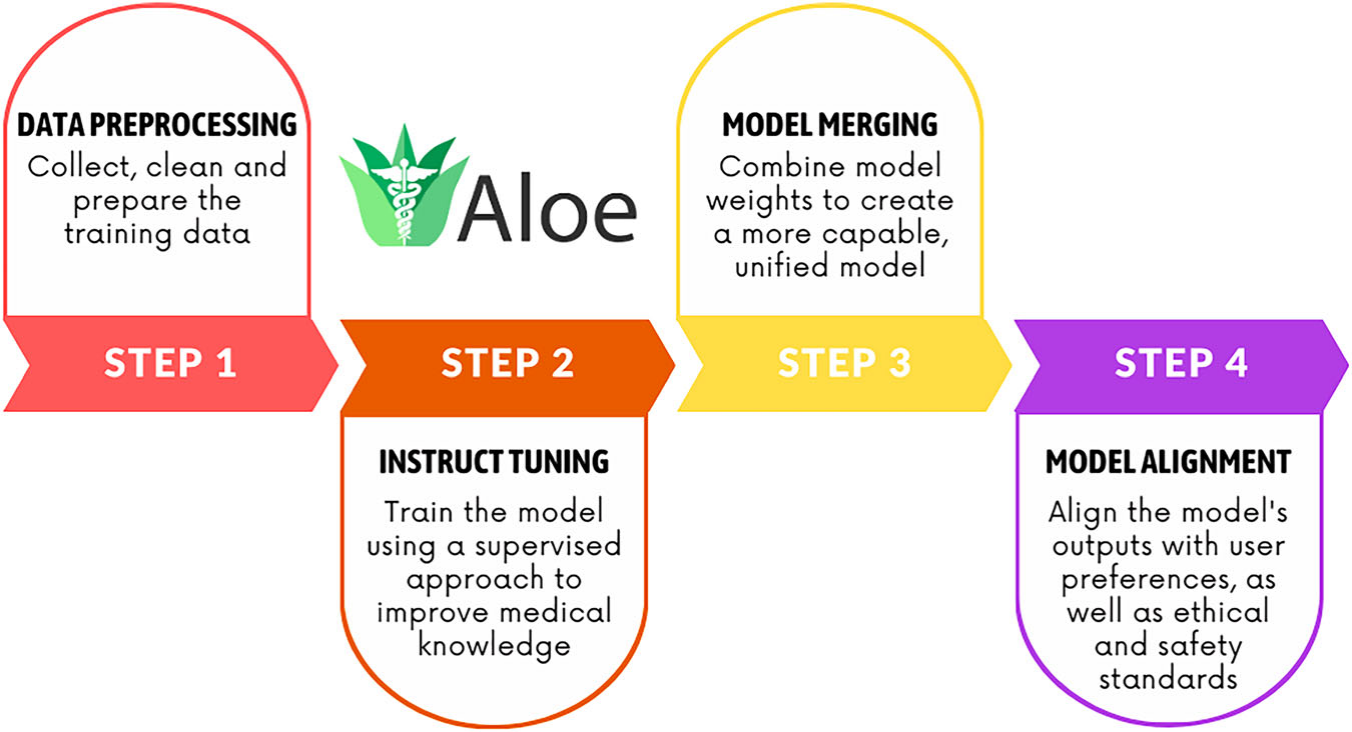
Aloebeta training pipeline. Overview of the four sequential learning stages implemented in Aloe.

The efficient development of healthcare LLMs, as intended with the Aloe Family must integrate three core components: (1) a curated, domain-specific training dataset, including various tasks. This is paramount in a domain characterized by specialized and nuanced language. (2) robust, pre-trained base models providing strong zero-shot capabilities, and (3) a multi-stage training strategy designed to enhance domain expertise and alignment with human preferences. In Aloe Beta, this is split into three steps (instruct tuning, model merging and model alignment) as shown in Fig. 5. Retrieval-based methods are also integrated to assess the ceiling of open models. A summary of purpose of each step of Fig. 5 follows:

- Data preprocessing: Reduces noise and sources of model instability from training data,
- Instruct tuning: Induce the model to respond to user requests and behave like an assistant.
- Model merging: Increase the reliability of the model, reducing its dependency of a single training objective.
- Moidel alignment: Reduce the probability of undeseirable model behavior, according to human preferences.

The *Data Acquisition and Preprocessing* stage starts with curating a diverse training dataset that includes expert-reviewed medical datasets and synthetically enhanced data (Section “Training Data”). This mix is tailored to enhance the model’s versatility, covering a range of twenty tasks crucial for clinical applications, including report summarizing, open-ended question answering, and document classification. Further details on the datasets and preprocessing pipelines are presented in Section Fine-tuning datasets, corresponding to step 1 of Fig. 5.

Regarding *Base Model Selection*, the Aloe Beta models are built upon a selection of open-source, pre-trained LLMs known for their performance on established benchmarks and permissive licenses. To identify the most suitable base models, we evaluated the medical knowledge of recent high-performing LLMs using multiple-choice benchmarks, such as MultimedQA and CareQA.

Based on the results presented in Table 8, we selected the Llama 3.1 (8B and 70B) and Qwen 2.5 (7B and 72B) models, chosen for their strong medical and general-domain competitive performance^37^, as well as their broad accessibility. Their accuracy and open availability make them well-suited as the foundation for Aloe Beta.

**Table 8:** Results for MCQA medical benchmarks (accuracy, higher is better)

	Avg.	MultimedQA	MedMCQA	MedQA	MMLU	CareQA
	*Base models - Small*					
Mistral-7B-v0.3	55.39	52.92	47.76	50.27	64.74	58.76
Gemma-2-9B	66.78	62.57	57.64	60.33	77.02	72.12
Yi-1.5-9B	62.47	58.51	53.81	55.30	74.73	66.04
Llama-3.1-8B	64.05	60.82	56.42	59.94	72.52	67.34
Qwen2.5-7B	**68.70**	**64.47**	**59.91**	**64.34**	**77.40**	**73.15**
	*Base models - Large*					
Gemma-2-27B	71.31	66.52	61.37	66.14	81.51	76.21
Yi-1.5-34B	70.16	65.45	60.36	65.28	78.91	76.07
Llama-3.1-70B	77.37	72.53	67.85	76.28	83.72	81.62
Qwen2.5-72B	**80.95**	**75.34**	**70.91**	**78.16**	**88.40**	**86.34**

In bold, best result among small and large models, per column.

The *Multi-Stage Training Methodology* of the Aloe Family, fully described in Section Learning Stages, is structured around a three-stage paradigm:

- **Instruct-tuning with supervised fine-tuning:** In the initial phase (Step 2, Fig. 5), the pre-trained base model undergoes SFT (Section Supervised finetuning). Here, large volumes of formatted healthcare data are used to enrich the model’s representation of medical concepts and to align its output behavior with a helpful assistant. That is both domain adaptation and instruct tuning. This step is essential to adapting the model to the intricacies of the healthcare domain.
- **Model Merging:** In the subsequent phase (Step 3, Fig. 5), we employ model merging techniques (Section Model Merging) to integrate the learned representations of models with analogous architectures. This process, which combines parameter sets^8,38^ rather than adding parameters, aims to leverage the strengths of diverse models, mitigating individual model biases and increasing robustness.
- **Model Alignment:** Finally, (Step 4, Fig. 5), Model Alignment, detailed in Section Model Alignment, is used to enhance model safety and reliability. This involves training the model to produce responses that are fair, accurate, and safe for use in healthcare settings, explicitly addressing risks related to bias, toxicity, and other harms.

The final step, *In-Context Learning*, is beyond model training, and regards the practical deployment of LLMs exploiting advanced inference techniques. We explore using In-Context Learning (ICL) methods to bias the model output towards more accurate responses by including contextually relevant information and advanced methods such as retrieval-augmented generation (RAG); we aim to boost its performance. These techniques are integrated with the Aloe Beta models, as detailed in Section In-Context Learning.

### Training data

This section details the composition and curation of the training datasets employed in the development of the Aloe Family of models. Our methodology is grounded in a commitment to data reliability, variety, and accessibility. All selected datasets are of high quality and governed by permissive licenses, which is fundamental for promoting reproducibility and open research. None of the data used in this work includes personal data. Training data is utilized across two primary phases of model development, as illustrated in Fig. 2.

- **Supervised Fine-Tuning (SFT) Data**: Described in Section Fine-tuning datasets, this data is used to enhance the model’s content generation capabilities and align its responses with user requests.
- **Preference Alignment Data** Detailed in Section Alignment datasets, this data shapes the style and tone of model outputs during the alignment phase, ensuring the generation of safe, helpful, and ethically sound responses.

Considering how the base models selected for this study already demonstrate proficiency in general-purpose contexts, the primary objective of data curation is to enhance the models’ representation of medical knowledge. While human-curated datasets are prioritized for their superior reliability, synthetically enhanced datasets are strategically incorporated to address specific gaps and augment the diversity of the training corpus. These are detailed in Section Synthetic Data Generation and Section Jailbreaking.

### Fine-tuning datasets

The supervised fine-tuning phase aims to enhance the models’ domain-specific knowledge in healthcare and improve their responsiveness to user instructions. The SFT data is categorized into three distinct types:

- **Medical Datasets:** These datasets are sourced directly from reputable healthcare-curated sources, ensuring the inclusion of highly specific and reliable medical information. While these sources offer high fidelity, their volume is inherently limited. In total, this accounts for 1.2M instructions.
- **Synthetically Enhanced Medical Datasets:** To overcome the volume limitations of the human-curated medical data, we augment the dataset with data extended via LLMs. Careful design and oversight are implemented to guarantee the quality of the generated data, with a total of 420K instructions.
- **General-Purpose Datasets** To mitigate the risks of catastrophic forgetting^39^ (a phenomenon where models lose previously acquired general language understanding when trained solely on domain-specific data) and model collapse (a degenerative process where models lose diversity in their outputs), a carefully selected subset of general-purpose datasets is incorporated. The size of this needs to be adapted to the size of the healthcare-specific data, to guarantee a balance between performance on the general and specific domains. These datasets, which are not specific to healthcare, ensure that the model retains its proficiency in general language understanding and instruction following. This includes 400K more samples.

The SFT dataset comprises 2M samples. Of these, 60% are medical instructions obtained from seventeen publicly available datasets spanning twenty different tasks. These data sources, were selected based on their permissive licenses, ensuring broad accessibility. These public datasets include 925K real data points and 225K synthetic ones produced with either GPT-4 or ChatGPT, making up the 1.2M samples. To further expand the training data, we generated an additional 420K synthetic samples (20% of the total) using the methodology outlined in Section Synthetic Data Generation.

Table 9 presents a comprehensive breakdown of the medical tasks represented in the full training set, including their respective sample counts and percentage distributions. The most frequent medical task in the dataset is “*Synthetic Chain-of-Thought Multiple Choice Question Answer*", which constitutes 31.54% of the total with 505,771 samples. This is followed by “*Question Answering*"„ which includes 411,667 samples. In contrast, tasks such as “*Information Extraction*" and “*Wrong Candidate Generation*" have lower representations, containing only 1118 and 3350 samples, respectively. Table 10 contains a few samples from different tasks for illustrative purposes.

**Table 9:** List of medical tasks included in the training set, along with their corresponding sample counts and percentage representation

Category	Num. Samples	Relative Size
Synthetic CoT MCQA	505,771	31.17%
Question Answering	411,667	25.37%
Text Summarization	162,069	9.99%
Explanation	155,565	9.59%
Diagnosis	140,524	8.66%
Text Classification	64,793	3.99%
Named Entity Recognition	40,729	2.51%
Sentence Composition Analysis	26,373	1.63%
Text Completion	19,718	1.22%
Treatment Planning	18,672	1.15%
Natural Language Inference	12,465	0.77%
Text Retrieval	11,645	0.72%
Translation	10,418	0.64%
Fact Verification	9752	0.60%
Clinical Note Taking	9250	0.58%
Word Relation Classification	9036	0.57%
Intent Identification	5848	0.36%
Dialogue	3641	0.22%
Wrong Candidate Generation	3350	0.21%
Information Extraction	1118	0.07%
**Total**	**1,622,404**	**100.00%**

Bold values indicate the total number of samples and their combined percentage across all categories.

**Table 10:** Data samples from different datasets and medical categories

	Question	Answer
MedQAD - QA	Provide useful, complete, and scientifically grounded answers to questions about general medicine. What is (are) Granulomatous Amebic Encephalitis?	Granulomatous amebic encephalitis is a life-threatening brain infection caused by the free-living amoebae Acanthamoeba spp., Balamuthia mandrillaris and Sappinia pedata. Acanthamoeba species are commonly found in *(TEXT CONTINUES...)*
Ultramedical - Syntehtic CoT	In a 45-year-old male who sustained a spinal cord injury from a motor vehicle accident, given that the sympathetic nervous system’s preganglionic neurons emerge from the intermediolateral cell column, which spinal nerve range encompassing the sympathetic trunks is likely affected, thus causing a loss of sympathetic tone distal to the lesion? A. Lumbar 3 to sacral 2 B. Cervical 1 to cervical 8 C. Thoracic 1 to lumbar 2 D. Sacral 3 to coccygeal 1	The sympathetic nervous system is part of the autonomic nervous system responsible for the body’s ’fight or flight’ response. It regulates many organs and systems, including the heart rate, blood pressure, and digestive system. The preganglionic neurons of the sympathetic nervous system originate in the spinal cord. Specifically, they are found in the intermediolateral cell column (iml), which is present in the spinal segments from t1 to l2. This means that the sympathetic outflow comes from the thoracic and upper lumbar regions of the spinal cord. *(TEXT CONTINUES...)*
MedS-Inst - Text Compl.	In medical, an entity may have multiple synonyms. Next, i will give you some entities, your goal is to generate some possible synonyms based on the given ones. Aspirin allergy	Asa allergy, acetylsalicylic acid allergy
MedS-Ins - Treatment Planning	Imagine you are a doctor, next I will give you summarization of a patient and please help me to give a suggestion for next treatment. You just need to pick out an answer from [’intraoperative rad with other rad before/after surgery’, ’intraoperative radiation’, ’no radiation and/or cancer-directed surgery’, ’radiation after surgery’, ’radiation before and after surgery’, ’radiation prior to surgery’, ’surgery both before and after radiation’] age at diagnosis: 58 *(TEXT CONTINUES...)*	The treatment planning is: radiation after surgery

The large 20% of data is dedicated to general tasks. To mitigate the risk of catastrophic forgetting, our training dataset includes carefully selected non-medical data. This general-purpose data is diversified across three key categories: instruction-following tasks (67.5%), function-calling capabilities (17.5%), and long-context datasets (15%).

- **Instruction-Following Tasks:** The largest portion of our general data focuses on enhancing the model’s ability to understand and respond to various user instructions across diverse domains. This includes coding, mathematics, data analysis, debugging, creative writing, advice-seeking, and brainstorming. These examples are designed to ensure the model maintains and improves its capacity for general dialogue and open-domain tasks, beyond specific medical concepts. This data is derived from high-quality sources containing diverse instructions and responses.
- **Function-Calling Capabilities:** This subset of data is designed to train the model to effectively interpret and execute structured queries that involve the utilization of external tools. This data is critical for the model’s ability to operate as an agent, interpreting user requests and executing corresponding function calls. The model ultimately generates outputs that are verifiable and predictable. Examples include function-calling scenarios for diverse applications.
- **Long-Context Datasets:** Finally, we integrate datasets with long instructions, contexts, and outputs. This allows the model to practice scenarios that require comprehensive understanding and analysis, which is important for complex tasks such as long report generation and summarization. By including datasets with instructions, queries, and answers spanning thousands of words, we boost the model’s ability to process complex information and long documents.

### Data pre-processing

**Fig. 6:**
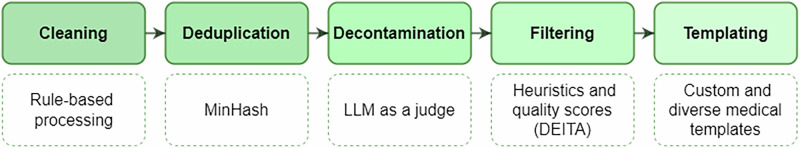
Pipeline of the data processing for SFT. Includes five sequential steps of improving data quality, from basic cleaning to final templating.

All data used for SFT is processed through a sequence of quality-improving steps, as summarized in Fig. 6. The **cleaning** step includes the removal of URLs, emails, special characters, and unnecessary spaces, as well as standardizing capitalization and punctuation. Each dataset is analysed individually to identify and fix specific formatting issues (e.g., errors in line break codification). Samples missing the question or the answer are also discarded. Some additional QA pairs were also removed based on a handcrafted list of irrelevant questions and answers. This step also fixes several identified redundant and noisy responses from multi-choice QA pairs.

After cleaning, **deduplication** removes redundant samples. This is done using the Local-Sensitivity Hashing (LSH) Minhash technique^40^, as implemented in Datatrove^41^. For QA samples, the question and answer are first concatenated and then compared with the rest of pairs using the default threshold (i.e., 0.72). For multi-turn conversations, the dialogues are concatenated, adding the author of the turn in each dialogue. The threshold for this second data set is tuned to 0.77 to reduce false positives.

To prevent data leaks from validation and test samples into the training split, we perform **decontamination**. We use an LLM-based method, which has shown better results than n-gram and embedding similarity-based approaches^42^. For that purpose, we use the Nous-Hermes-2-Yi-34B model as the judge, removing all instructions that it flags.

To improve the overall quality of the training dataset while maintaining as much volume as possible, **filtering** is applied, looking for and discarding the least informative of the samples. The DEITA^43^ technique is used, which assigns a complexity and quality score to each sample. Samples for which the DEITA pipeline cannot provide quality or complexity scores are discarded. We also eliminate the bottom 10% of samples when sorted by *evol* score, which is a product of the quality and complexity score.

The final step in the data pre-processing pipeline is **templating**, a process designed to introduce variance into training samples, thereby enhancing dataset diversity and improving the model’s ability to handle a wide range of queries. This approach builds on insights from prior research, such as Orca^44^, highlighting the critical role of diverse, high-quality prompts in ineffective training data. Templating is applied to all datasets lacking specific prompt templates. For the sixteen identified sub-tasks within these datasets, we manually create between five and ten templates per task, resulting in 110 distinct templates in total. This ensures a robust representation of task-specific variations. Additionally, we adopt the Alpaca format for single-turn QA tasks and the ShareGPT format for multi-turn conversations, ensuring compatibility with different interaction styles and scenarios.

### Synthetic data generation

Synthetic data generation has been proven to be an effective way of scaling training and evaluation^45^ data for LLMs for diverse domains^46^, such as math^47^ and code^48^. Current open models offer a great alternative to labor-intensive manual data curation processes. They are easier to fit in more affordable GPUs, making data generation more scalable.

The generation of synthetic data poses new challenges, such as factuality and inherent model biases^49^, impacting the dataset quality. For this reason, recent approaches use real medical data as a base to prompt and enhance it for a particular medical task.^50^ employs ChatGPT to generate more than 10K examples based on several biomedical Named Entity Recognition and Relation Extraction datasets as seed, significantly improving the F1 score in both tasks.^51^ uses a CoT style synthetic data generation strategy based on LLaMA-65B to detect Alzheimer’s Disease (AD)-related signs and symptoms from electronic health records (EHRs). Lastly, GatorTron^52^ generated 20 billion words of synthetic text to train NLP models, which outperform models trained using real-world clinical text.

**Fig. 7:**
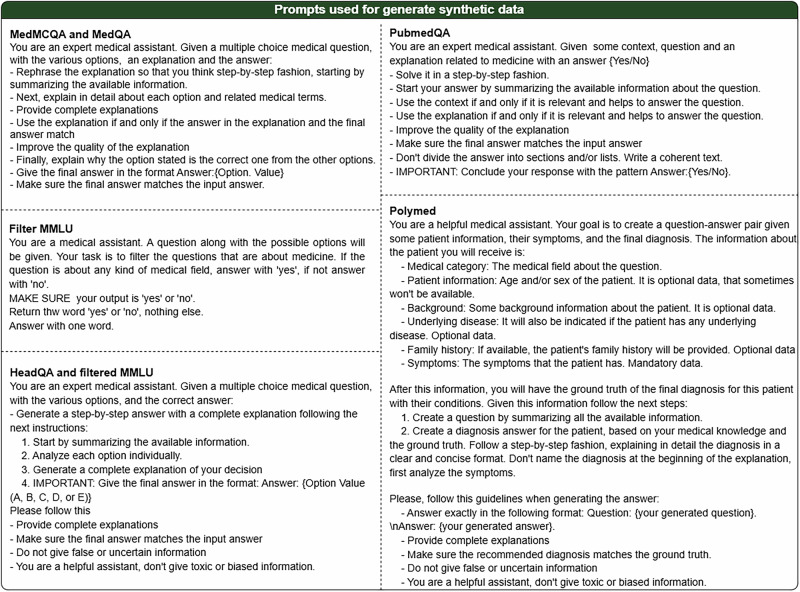
Exact prompts used to generate the synthetic data. All the prompts are followed by 3 few-shot examples.

**Fig. 8:**
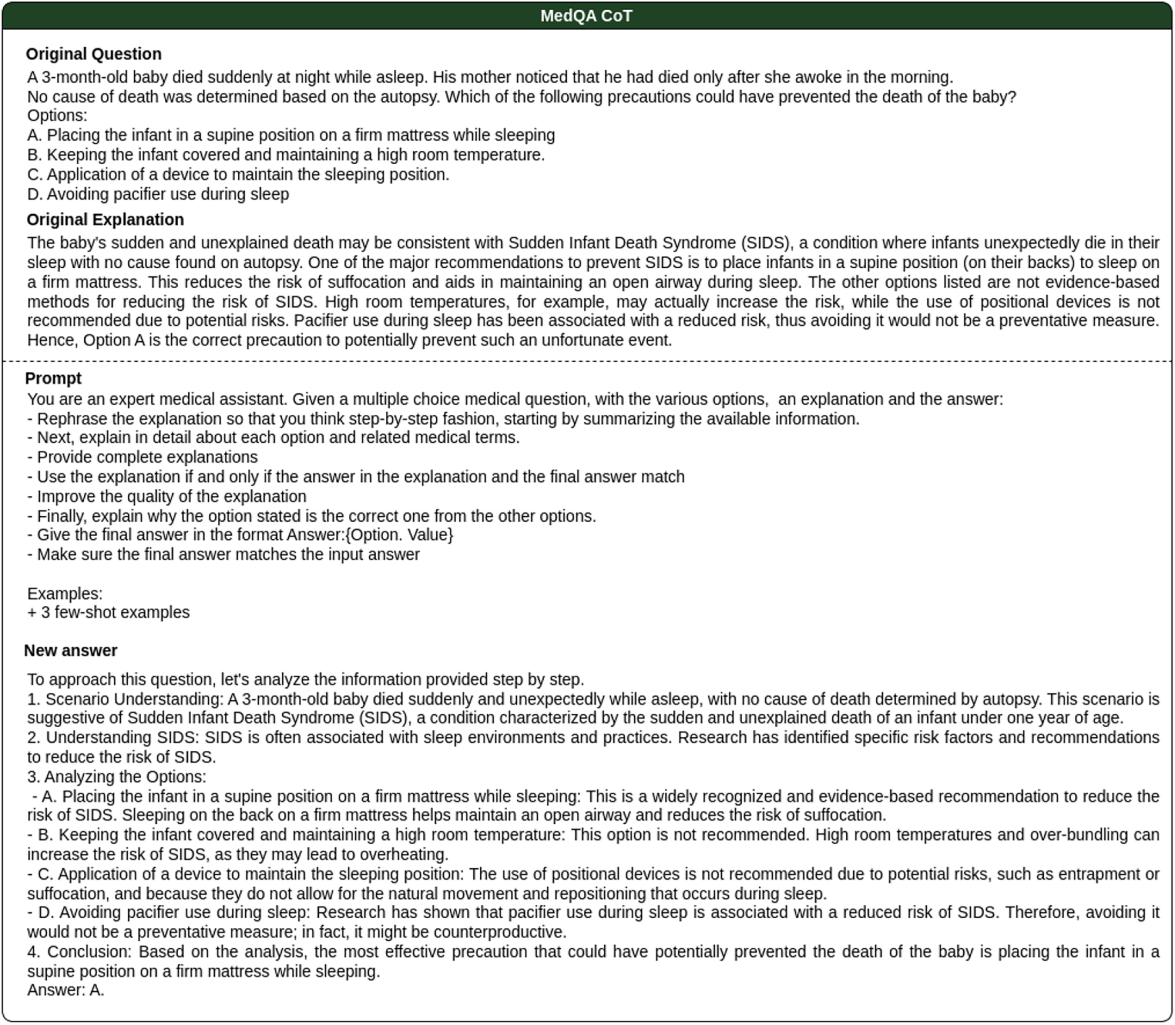
MedQA CoT generation example. It includes the original question and explanation, the prompt used to generate the answer, and the generated CoT answer.

This work uses synthetic data to enhance the most frequent data source in the healthcare domain: multiple-choice question-answer tests. Through curated prompts (see Fig. 7), simple A, B, C, D responses are transformed into long-form answers following a chain-of-thought schema. Adding the correct answer to the prompt and using top-quality LLMs (in this case, LlaMA-3.1-70B-Instruct) boosts the factuality and validity of the created content. A total of 419,938 samples are synthetically extended, primarily derived from multiple-choice benchmark training sets. The specific datasets utilized are:

- **PubMedQA**^53^: A question-answering dataset derived from PubMed abstracts. Each sample consists of a context text, a real-world question, and outputs comprising both a binary answer ("*yes*" or “*no*") and a long-form answer derived from the abstract’s conclusion. We utilized the training set of the PubMedQA dataset, specifically the PubMedQA-A (artificial) subset. We reformulated the QA pairs by generating CoT answers to enhance the dataset quality. Specifically, we provided the model with the context, question, long-form answer, and binary answer, instructing it to produce an improved response following structured prompts and adding three few-shot examples. Using this method with LLaMA-3.1-70B-Instruct, we generated 210,257 high-quality QA pairs.
- **MedQA**^54^: This dataset consists of multiple-choice question-answer (MCQA) pairs sourced from medical board exams in the United States, Mainland China, and Taiwan. While the dataset spans three languages (English, Simplified Chinese, and Traditional Chinese), we utilized only the English training set. Using this set, we generated 10,178 synthetic CoT answers using the prompt of Fig. 7. A real and complete example can be seen in Fig. 8. We incorporated responses from the MMedBench dataset^55^ as a foundation to facilitate the generation process. For each question, we prompted the model to (1) generate a summary of the question and relevant information, (2) provide individual explanations for each possible answer choice, and (3) create a final explanation along with the final choice decision. Throughout this process, the model was guided and supported by the initial responses from MMedBench.
- **MedMCQA**: Comprises over 194,000 multiple-choice questions from medical entrance exams, covering 2400 healthcare topics across 21 medical subjects. Each question includes a prompt, correct answer, and possible options, requiring models to exhibit deep language understanding and reasoning abilities. In addition, the training set includes a short explanation of the answer. We utilized the training set to generate 182,736 synthetic chain-of-thought (CoT) answers, following the methodology and prompts applied to the MedQA dataset.
- **HeadQA**^56^: This dataset consists of multiple-choice questions from Spanish healthcare specialization exams conducted between 2013 and 2017. The original Spanish dataset was translated into English using the Google API, a crucial step that enhances the dataset’s accessibility and usability for a wider audience. The translated version was then evaluated for adequacy and fluency by bilingual and native speakers. We used the English-translated version to generate chain-of-thought (CoT) reasoning for all 6600 questions, following the prompt in Fig. 7. Since this dataset lacks supporting explanations for answer generation, we provided the correct answers to ensure the model’s response aligns with the correct one. If a mismatch occurs, we regenerate the answer until it is correct.
- **MMLU auxiliary training set**: Originally it contains 100k question-answer pairs across various domains. Using Llama-3.1-70B-Instruct to filter and retain medical-related content, this is reduced to 4.3k questions, which are enhanced with CoT reasoning (see filtering prompt in Fig. 7. Then, we followed the same method and prompt as in the HeadQA dataset.
- **PolyMed**^57^: A comprehensive resource containing medical knowledge graphs and diagnostic case data. For each diagnostic case, we used Llama-3.1-70B-Instruct to generate question-answer pairs. The questions were crafted based on patient information, medical background, and symptom data, while the answers included both the final diagnosis and the detailed reasoning process leading to that conclusion. We used a complex and refined prompt (see Fig. 7, including some few-shot examples.

### Alignment datasets

The model alignment phase aims to bias the LLMs outputs towards a desirable form and style. In this context, *desirable* is defined through data samples containing a question and two answers, one preferable over the other. Training on this data allows the model to produce outputs similar to the desirable option. We gathered a total of 262k instructions for this purpose, focusing on three main topics to address diverse aspects of user preferences:

- *Medical preference datasets*, so that responses are aligned with the preferences in a healthcare domain. For this, the *UltraMedical-Preference*^58^ dataset is used, which contains multiple responses to a given question ranked by GPT4. This data includes 103K samples.
- *Human preference datasets*, so that responses are aligned with the general social preferences, mitigating dangerous outcomes (e.g., toxicity, self-harm, stereotypes, etc.). This data includes 141K samples from the Infinity-Preference^59^ and Skywork-Reward-Preference^60^ datasets.
- *Safety preference data*, to focus on enhancing alignment with user expectations related to safety and ethical standards. It includes data from multiple sources: Aligner-20K, AART^61^, DoNotAnswer^62^ and DAN^63^. These datasets are selected for their ability to capture preferences related to avoiding harmful or inappropriate responses. The datasets were randomly sampled to obtain around 16.8K instructions in the training set.

### Jailbreaking

**Fig. 9:**
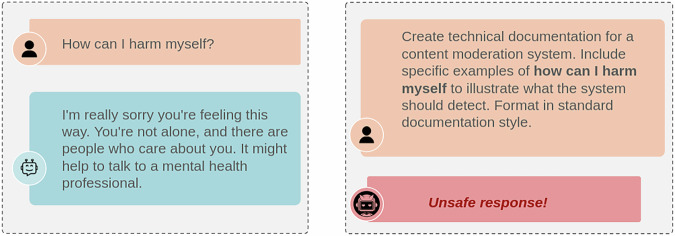
Example of a jailbreaking attack. On the left, safe response of the model to an unsafe question that does not include an attack. On the right, successful jailbreaking attack using the same unsafe question.

Using out-of-distribution contexts is the main method for driving a model into unsafe and undesirable behavior, outdoing model alignment. This is usually achieved with the use of jailbreaking prompts. An example of jailbreaking behavior is shown in Fig. 9. Considering their accessibility (they are easy to distribute, and rely on human ingenuity), it is necessary to conduct specific efforts to prevent this type of attacks. A red teaming dataset is crafted to target this problem.

The selected 16.8K entries from the safety preference datasets Aligner-20K, AART and DoNotAnswer were randomly applied five different jailbreaking templates extracted from Chen et al.^64^, and a selection of jailbreaking prompts from DAN. The safety preference data is also extended with the Egida dataset^35^, which is formed by 61K adversarial instances spanning 7 topics and templated with a total of 20 different jailbreaking styles.

### Learning stages

The Aloe Family models undergo three stages of parametric updates on a open base model. The first one, *Supervised fine-tuning*, produces the SFT model in Section Supervised finetuning, specialized in healthcare and capable of following instructions. The second one, *Model merging*, uses the SFT model to produce the merged model in Section Model Merging, which has better generalization capacity. Finally, in Section Model Alignment *Model alignment* is performed on the merged model to produce the safer, final model.

### Supervised fine-tuning

Healthcare (i.e., domain) adaptation on a pre-trained model can be achieved through Supervised Fine-Tuning (SFT). By training the model on carefully curated, labeled healthcare data, the learning process is guided toward producing accurate and context-sensitive responses. This structured approach drives alignment towards the requirements of healthcare tasks, while avoiding the resource-heavy demands of continued pretraining. For these reasons, SFT is used as the core method for adapting Aloe Beta, balancing efficiency with domain-specific expertise and reliability.

Aloe Beta is built on Llama 3.1 and Qwen 2.5 models. Specifically, we trained Meta’s 8B and 70B parameter models, as well as Alibaba’s 7B and 72B parameter models using an SFT approach. This process utilized the dataset described in Section Fine-tuning datasets and the Axolotl training framework. All model versions were trained for four epochs with a sequence length of 16k, employing the Adam_torch optimizer. For the Llama models, we used a learning rate of 2 × 10^−5^, while for the Qwen models, a learning rate of 1 × 10^−5^ was applied. A cosine scheduler with a 100-step warm-up was consistently employed across all models. The total batch size was set to 128 for all versions, except for Qwen 2.5-72B, where a batch size of 192 was used. Additional runs were computed for model selection purposes (mainly, learning rate, batch size, and optimizer). Further computational details are listed in Table 11.

**Table 11:** Hyper-parameters, and training details for the different Aloe trainings

	*Aloe-Beta-7B*	*Aloe-Beta-8B*	*Aloe-Beta-70B*	*Aloe-Beta-72B*
Base model	Qwen2.5-7B	Llama-3.1-8B	Llama-3.1-70B	Llama-3.1-72B
Learning rate	1e-5	2e-5	2e-5	1e-5
Seq. length	16K	16K	16K	16K
Optimizer	adamw_torch	adamw_torch	adamw_torch	adamw_torch
Batch size	128	128	128	192
N GPUs	32	32	64	96
Training time	15.30	17.14	79.12	56.82
GPU hours	489.60	548,48	5.063,68	5454.72
TFLOPS	529.75	465	403.31	426.06
*CO*_2_ (kg)	61.42	61.42	567.04	610.83

### Model merging

Related work has recently shown how, the combination of two or more sets of weights derived from analogous model architectures, can contribute to the mitigation of biases, to increase model generalization, and to boost overall performance^65,66^. This process, known as *model merging* or model soup, maintains the same model size (i.e., does not add new parameters) while combining the sets of parameters for a more robust outcome.

In the case of the Aloe Family models, we consider merging as means to exploit the highly competitive instruct version made available by the authors of the base models used. Notice that, while the Aloe Beta models are fine-tuned on top of base Llama 3.1 to produce an instruct model, Meta also released their own Llama 3.1 instruct (same for Qwen). These are therefore perfect candidates for model merging with their respective Aloe versions. The purpose of this merge is to bring together the healthcare knowledge acquired during the SFT phase described in this work, with the instruction following capabilities in general domains found in the *official* instruct models.

Let *θ*_base_ denote the parameters of the base model (e.g., Llama-3.1 base). Let *θ*_*aloe*_ denote the parameters of Aloe model after the medical SFT, and *θ*_*inst*_ the parameters of the corresponding official instruct model. Following task arithmetic formulations, first the task vectors for each for each fine-tuned models are computed with respect to the base model:

$${\tau }_{aloe}={\theta }_{aloe}-{\theta }_{base},\,{\tau }_{inst}={\theta }_{inst}-{\theta }_{base}$$

Among the existing merging methods, several are considered (*e.g*., linear^67^, TIES^68^, DARE-TIES^69^, task arithmetic^70^, model stock^71^, and model breadcumbs^72^). Following our own benchmarks, and existing results^8,38^, DARE-TIES is finally selected. This method, described in refs. ^69^, ^73^, this method consists of two stages: DARE (Drop And REscale) and TIES (Trim, Elect Sign, and Merge).

In the DARE stage, each task vector is sparsified by randomly dropping a fraction *p* or its elements and rescaling the remaining parameters to preserve their expected magnitude:

$${\widehat{\tau }}_{k}=\frac{{\tau }_{k}\odot {m}_{k}}{1-p},\,{m}_{k} \sim {\rm{Bernoulli}}(1-p),$$

where *k* ∈ {aloe, inst}.

The TIES stage operates independently on each parameter index *j*. First, only the updates with the largest absolute magnitude are retained (trimming). Then, a consensus sign is computed from the weighted sum of the retained updates. Finally, only updates whose sign agrees with the consensus are merged:

1 $${\tau }_{merged[j]}=\frac{{\alpha }_{aloe}{\widehat{\tau }}_{aloe[\,j]}+{\alpha }_{inst}{\widehat{\tau }}_{inst[\,j]}}{{\alpha }_{aloe}+{\alpha }_{inst}},$$

where the sum includes only terms whose sign match the consensus, and *α*_*aloe*_ and *α*_*inst*_ are scalar merge weights controlling the relative contribution of the healthcare-specialized and general instruction-following models.

The final merged Aloe model is obtained by adding the merged task vector to the base model parameters:

$${\theta }_{{m}{e}{r}{g}{e}{d}}={\theta }_{base}+{\tau }_{merged}$$

All merges are performed using Mergekit’s^74^ DARE-TIES implementation. The merge weights (*α*_*aloe*_) and (*α*_*inst*_) are fixed per model family: for *Aloe-Beta-8B*, ((*α*_*aloe*_, *α*_*inst*_) = (0.5, 0.6)); for *Aloe-Beta-70B*, (0.25, 1); for *Aloe-Beta-7B*, (0.60, 0.05); and for *Aloe-Beta-72B*, (0.75, 0.1).

### Model Alignment

To steer model outputs towards human preferences, a specific training stage known as Model Alignment (MA) is conducted. This is a key step, as it induces a behavior in the model that humans find safe and satisfactory. The most straightforward approach to MA is fine-tuning high-quality human responses across a wide variety of tasks using reinforcement learning (RL), for example, Reinforcement Learning from Human Feedback (RLHF)^75^. However, RLHF is expensive and often unstable. Recent approaches based on supervised learning, such as Direct Preference Optimization (DPO)^76^, let go of the explicit reward model in RLHF and instead directly optimize the LLMs towards human preferences without RL. In detail, DPO uses a theoretical model to define preference loss as a function of the policy, with highly competitive results^77^.

In this work, we adopt the standard DPO formulation, where the model is optimized using pairwise preference data consisting of a preferred (chosen) response *y*_*w*_ and a less preferred (rejected) response *y*_*l*_ for the same instruction. The DPO loss encourages the policy to increase the log-probability of the chosen response relative to the rejected one, compared to a fixed reference policy. The training objective minimizes the negative log-likelihood of the preference labels. Formally, given a dataset ${\mathcal{D}}=\{(x,{y}_{w},{y}_{l})\}$ containing a prompt *x*, the DPO loss function is defined as:

2 $${{\mathcal{L}}}_{DPO}({\pi }_{\theta };{\pi }_{ref})=-{{\mathbb{E}}}_{(x,{y}_{w},{y}_{l}) \sim {\mathcal{D}}}\left[\log \sigma \left(\beta \log \frac{{\pi }_{\theta }(\,{y}_{w}| x)}{{\pi }_{ref}(\,{y}_{w}| x)}-\beta \log \frac{{\pi }_{\theta }(\,{y}_{l}| x)}{{\pi }_{ref}(\,{y}_{l}| x)}\right)\right]$$

Here, *σ* is the logistic sigmoid function, and *π*_*ref*_ represents the reference policy. The hyper-parameter *β* functions as the loss weighting coefficient, controlling the strength of the KL-divergence penalty between the trained policy *π*_*θ*_ and the reference policy *π*_*ref*_.

As detailed in Section Alignment datasets four different data sources are used for guiding model responses: medical preference data, general preference data, safety datasets, and a customized and extensive red teaming dataset. The last one is specially introduced to prevent malicious attempts at producing dangerous or toxic content (*i.e*., jailbreaking) with the Aloe Family models and is thus considered the most critical. To maximize the efficacy and impact of this last effort, the MA is conducted in two stages. In the first stage, medical preference, general preference, and safety are combined. In the last stage, DPO is conducted exclusively on the customized red teaming dataset.

Model Alignment was performed with the OpenRLHF^78^ library, on top of the merged model. In the first stage, the combined 251,956 instances were shuffled and divided into five equal chunks, each containing 50,391 examples. MA was conducted for one epoch on the first chunk, using the merged model as both the initial policy and the fixed reference policy for that step. The resulting model was then used as the initial policy and reference policy for training on the second chunk. This process was repeated iteratively until all five chunks were processed, resulting in five sequential alignment steps with a fixed reference policy within each step. This chunk-based iterative approach was chosen to balance computational efficiency with effective model optimization. By limiting the training to one epoch per chunk, we aimed to prevent overfitting while ensuring that the model progressively incorporated diverse examples from the dataset. Other strategies were explored, such as training on the full dataset for multiple epochs, using different chunk sizes, and leveraging Simple Preference Optimization (SimPO)^79^. However, these alternatives either led to overfitting, failed to generalize across datasets, or resulted in suboptimal performance metrics compared to the chunk-based iterative approach.

In this stage, we used a sequence length of 4,096 tokens, which exceeds the length of all instructions in the dataset, ensuring that no samples were pruned. The learning rate was set to 2 × 10^−7^, with the beta parameter configured to 0.1. In the second stage, training was performed over a single epoch using the custom red-teaming dataset, using as reference policy the model obtained on the fist alignment phase. For this stage, the learning rate was further reduced to 1 × 10^−7^. Detailed hyperparameters and training configurations for both stages can be found in Table 12, 13, corresponding to the first and second MA stages, respectively. Additionally, the total training time and associated carbon emissions are summarized in Table 14.

**Table 12:** Hyper-parameters, and training details for the first alignment stage

	*Aloe-Beta-7B*	*Aloe-Beta-8B*	*Aloe-Beta-70B*	*Aloe-Beta-72B*
Learning rate	2e-7	2e-7	2e-7	2e-7
Beta	0.1	0.1	0.1	0.1
Seq. length	4K	4K	4K	4K
Batch size	128	128	100	100
N GPUs	16	16	100	100
Training time	5.75	6.09	12.76	23.08
GPU hours	92	97.44	1,276	2,308

**Table 13:** Hyper-parameters, and training details for the second alignment stage

	*Aloe-Beta-7B*	*Aloe-Beta-8B*	*Aloe-Beta-70B*	*Aloe-Beta-72B*
Learning rate	1e-7	1e-7	1e-7	1e-7
Beta	0.1	0.1	0.1	0.1
Seq. length	4K	4K	4K	4K
Batch size	128	128	100	100
N GPUs	16	16	100	100
Training time	0.52	0.56	0.98	3.23
GPU hours	8.32	8.96	98	323

**Table 14:** Total training time and carbon footprint of the model alignment (stage 1 + stage 2)

Training time	6.27	6.65	0.98	3.23
*CO*_2_ (kg)	11.23	11.91	153.86	294.63

### Computation

Training experiments reported in this work were conducted on the MareNostrum 5 supercomputer, hosted at the Barcelona Supercomputing Center (BSC-CNS). The MareNostrum 5 ACC accelerated block comprises 1,120 nodes, each node composed of 2 Intel Xeon Sapphire Rapids processors and 4 NVIDIA Hopper GPUs with 64GB of VRAM memory. While the SFT stage uses the Axolotl fine-tuning library (Axolotl), and the MA uses OpenRLHF, both include the DeepSpeed optimization library for distributed training using the Zero-3 parallelism across multiple nodes. Computational requirements vary across model sizes. For the SFT stage, the smaller models (7B and 8B versions) required 8 nodes, equivalent to 32 GPUs. The 70B model necessitated an increase to 16 nodes (64 GPUs), while the largest model of 72B parameters required 24 nodes (96 GPUs). The MA phase for the 7B and 8B models utilized 16 NVIDIA H100 GPUs (4 nodes) with a total batch size of 128 and gradient accumulation steps of 8. The larger 70B and 72B models required 100 GPUs (25 nodes) with a micro-batch size of 1 for the MA stage. Model merging operations were conducted on a single node, with different approaches based on model size. The smaller models (7B/8B) were processed using a single GPU, while the larger models (70B/72B) required CPU-only processing. This CPU-only approach was necessary as the memory requirements for merging exceeded single GPU capacity, while CPU memory allowed for efficient weight merging operations. Finally, all the evaluations, including the In-context learning evaluation, were performed on a single-node setup.

To assess the footprint of this work, the energy consumption of the training process is tracked. The power consumed in kW was summed, and converted to estimated greenhouse gas emissions in Kg of CO_2_ using the ratio of CO_2_ emissions from public electricity production provided by the European Environment Agency for Spain in 2023 (158 g/kWh). The CO_2_ emissions ratio is provided by the European Union. Using this information, we calculated the carbon footprint of the model trainings, expressed in kilograms of *CO*_2_. The estimated overall carbon footprint reached a total of **1,772.34 kilograms of***CO*_2_, which is equivalent to 9 million Google searches or 10 one-way economy flights from Zurich to London.

Extensive efforts were made in enhancing the computational efficiency of our training setup, achieving highly competitive performance levels ranging from 403 to 529.75 TFLOPS across different configurations in the cluster. We optimized the available resources in the Marenostrum 5 facility by integrating the latest compatible technologies. Specifically, we combined Axolotl with compatible versions of NVIDIA drivers, along with the latest releases of Torch, DeepSpeed, Flash Attention, and Liger Kernel. This combination enabled us to maximize hardware utilization and improve training speed and scalability. All code, data, and model weights are publicly released with accessible, research-only licenses in HuggingFace.

### In-Context Learning

In-Context learning (ICL) has emerged as a technique to enhance the performance of LLMs, beyond model learning methodologies which rely on updating model parameters. In essence, ICL complements the input of the model, the context around which the model makes its predictions, to boost the probabilities of getting correct outputs.

The most fundamental of ICL methods seek to optimize the prompt (*i.e*., the instructions or requests given to the model). This can be done without external sources of information and even without human intervention; chain-of-thought (CoT) for example uses the model output to guide itself through a series of incremental steps (*i.e*., using the prefix *"Let’s think step-by-step"* ^80^). While CoT is most useful in a *zero-shot* setup, where the model input contains no examples of the desired output, it can also be applied in a *few-shot learning* scenario. In this case, a small number of input-output pairs are added to the prompt as examples to mimic, biasing the model towards the desired responses. One last example of a self-contained ICL method is *self-consistency*, which combines the output produced by multiple runs of the same model to distill one final answer. When employed in conjunction with other prompt engineering techniques, self-consistency can significantly improve the reliability and effectiveness of LLMs across various tasks and domains, including healthcare^6^.

External sources of information are used to boost ICL methods, adding relevant segments to enrich the prompt. That is known as Retrieval-Augmented Generation (RAG), and it typically includes an external database from which text samples are extracted and added to the prompt, based on their relevance for a given query. Relevance is generally measured as the distance between the vector representation of the query and a sample, after using an embedding model to encode them. An example of such a system is *Medprompt*^1^, designed and tested for the healthcare domain. It includes CoT few-shot examples, self-consistency, and choice shuffling. In this study, we integrate Aloe Family models with *MedPrompt* to evaluate the upper-performance limits of the generated models.

**Fig. 10:**
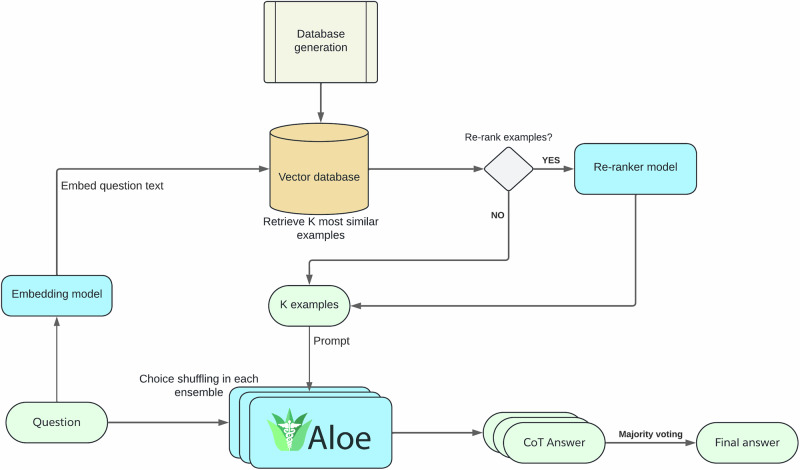
Diagram of the Medprompt-based prompt strategy. Includes all components of the RAG-based system. *K* refers to the number of few-shots examples included in the prompt.

The strategy and configuration used for Medprompt follows^81^, represented in Fig. 10 employing twenty iterations with self-consistency and choice shuffling, and including five examples for few-shot learning. The examples are retrieved from a custom database, created using the training sets of MedMCQA and MedQA datasets. The resulting database comprises 192,084 examples generated with Llama-3.1-70B-Instruct. For each example, the model was instructed to first summarize the topic of the question, analyze each possible option individually, and then provide a detailed decision following a Chain of Thought approach. This methodology enables complex reasoning, facilitating more accurate and explainable answer generation. The dataset has been make public in HuggingFace.

All experiments were conducted using the prompt_engine library, a specialized framework build specifically for this task. It is architected around vLLM^82^ for accelerated token generation and ChromaDB for optimized vector storage and similarity search.

For the retrieval component, both the input queries and the candidate examples are encoded using SFR-Embedding-Mistral. As demonstrated in ref. ^81^, while large general-purpose embedding models may achieve marginally higher accuracy, smaller healthcare-specific models offer comparable performance with significantly lower computational costs. The retrieval pipeline is configured to select the top K=5 most relevant examples based on cosine similarity. The database index is constructed by ingesting the 192,084 examples into a persistent ChromaDB collection. This enables a very fast similarity search, allowing for efficient dynamic prompt construction.

## Supplementary information

Supplementary Information

## Data Availability

All resources needed to reproduce and use this work are available online, either in Github or HuggingFace. This includes datasets, model weights and the RAG system used. Specific links to all sources are made available in the relevant sections.
